# Iridium(III) Complexes Bearing Pyrene- and Anthracene-Functionalized Ligands—Photophysics and Application Potential in Photocatalysis, Triplet-Triplet Annihilation Upconversion, Photodynamic Therapy, and Photoactivated Chemotherapy

**DOI:** 10.3390/molecules31111921

**Published:** 2026-06-02

**Authors:** Anna Kryczka, Katarzyna Choroba, Joanna Palion-Gazda, Barbara Machura

**Affiliations:** Institute of Chemistry, University of Silesia, 9 Szkolna, 40-006 Katowice, Poland; anna.kryczka@us.edu.pl (A.K.); joanna.palion-gazda@us.edu.pl (J.P.-G.)

**Keywords:** iridium(III), photosensitizers, photophysical properties, pyrene, anthracene, triplet-triplet energy transfer

## Abstract

Transition metal complexes that can serve as photosensitizers (PSs) have attracted considerable scientific interest owing to their potential applications in photodynamic therapy (PDT), triplet-triplet annihilation for energy upconversion (TTA UC), photocatalysis, and time-resolved bioimaging techniques. In many of these applications, the efficiency of intermolecular triplet-triplet energy transfer (TTET) between the photosensitizer and acceptor is largely determined by the triplet excited-state lifetime of the photosensitizer. One of the most efficient strategies for extending the triplet lifetimes of transition metal complexes is the incorporation of organic chromophores possessing long-lived intraligand (^3^IL) excited states into the coordination sphere of transition metal complexes. Polycyclic aromatic hydrocarbons, particularly anthracene- and pyrene-based chromophores, have emerged as especially attractive building blocks for this purpose. The current contribution highlights the role of pyrene and anthracene groups in controlling the photophysical properties of cyclometalated iridium(III) metal complexes, with an emphasis on their applications as photosensitizers. Particular attention is devoted to elucidating the relationships between molecular structure and excited-state properties. A detailed discussion of these relationships has been performed for three classes of cyclometalated iridium(III) complexes: (1) charge-neutral Ir(III) complexes including pyrene and anthracene motifs, (2) cationic bis-cyclometalated iridium(III) complexes bearing pyrene-functionalized ligands, and (3) cationic mono- and bis-cyclometalated iridium(III) complexes bearing anthracene-functionalized ligands.

## 1. Introduction

Since the pioneering work of Ford and Rodgers in 1992 [1], transition metal complexes bearing appended organic chromophores with long-lived triplet ^3^IL excited state have attracted significant scientific attention due to their outstanding photophysical behavior [2,3,4,5,6,7,8,9,10,11,12,13,14,15,16,17,18,19,20,21,22,23,24,25,26,27,28,29,30,31]. The integration of an organic chromophore possessing a long-lived intraligand (^3^IL) excited state with a transition metal complex molecule featuring an emissive triplet metal-to-ligand charge transfer excited state (^3^MLCT), typically characterized by a shorter emission lifetime (generally <1 μs), offers promising opportunities for tuning the photophysical properties of materials. The variations in photobehavior of such systems arise from the interplay between ^3^MLCT and ^3^IL states localized on an organic chromophore. When ^3^MLCT and ^3^IL excited states are energetically matched, photoexcitation can induce a reversible intramolecular triplet energy transfer equilibrium. In this case, an organic chromophore repopulates the luminescent ^3^MLCT excited state, serving as an energy “reservoir”, resulting in bichromophoric systems that exhibit prolonged ^3^MLCT emission lifetimes. Functionalization of transition metal complexes with *π*-conjugated groups may also result in intramolecular energy transfer from ^3^MLCT to a significantly lower-energy triplet state localized on the organic chromophore, leading to emission quenching. Such systems are non-emissive, but their lowest triplet excited states possess very long lifetimes. In rare cases, a dual emission originating from ^3^MLCT and ^3^IL excited states has been observed [2,3,4,5,6,7,8,9,10,11,12,13,14,15,16,17,18,19,20,21,22,23,24,25,26,27,28,29,30,31,32,33,34,35,36,37,38].

Elongated excited-state lifetimes and the ability to perform intermolecular triplet-triplet energy transfer (TTET) to other molecules make these systems appealing for employment in photocatalysis [39,40,41,42,43], triplet-triplet annihilation upconversion (TTA UC) [30,43,44,45,46,47,48,49,50,51,52,53,54,55,56,57], photodynamic therapy (PDT) [58,59,60,61,62,63,64,65,66,67,68,69,70], and photoactivated chemotherapy (PACT) [61,62,71,72,73,74,75,76,77]. The overall photosensitization efficiency depends on the absorption range and extinction ability of the photosensitizers, the intersystem crossing (ISC) efficiency, and the TTET process. The latter is determined by the triplet excited-state lifetime of the photosensitizer and the energy gap between triplet excited states of the photosensitizer and acceptor. The mechanism and efficiency of TTET have been comprehensively investigated both experimentally and theoretically [19,31,45,46,49,53,78,79,80,81,82,83,84].

Among the most recognizable organic chromophores employed in the bichromophoric approach are anthryl and pyrenyl groups, with triplet excited-state energies of approximately 2.06 and 1.8 eV, respectively [26,32,33,34]. Over the last four decades, numerous transition metal complexes incorporating anthryl- and pyrenyl-substituted ligands have been reported, and their photophysical properties have been extensively investigated, including the use of advanced experimental methods such as time-resolved infrared spectroscopy, fluorescence upconversion methods, time-resolved emission, and transient absorption spectroscopy. Several reviews have also summarized their photophysical characteristics and applications as photosensitizers [26,46,57,77,84,85,86,87,88].

To the best of our knowledge, a discernible gap in the current literature concerns the absence of a comprehensive review of cyclometalated iridium(III) complexes bearing pyrene (pyr) and anthracene (ant) functionalized ligands. Importantly, such systems can be regarded as outstanding candidates for the design of photosensitizers. This is attributed to the large spin–orbit coupling constant of iridium (3909 cm^−1^), which enables rapid and efficient intersystem crossing to long-lived triplet excited states, together with their high chemical and photostability, large Stokes shifts, tunable photophysical properties and ability to participate in intermolecular triplet-triplet energy transfer [19,30,44,45,46,48,52,53,58,64,74,78,79,80,87,89,90,91,92,93,94,95,96,97,98,99,100].

Therefore, a detailed discussion of the relationships between molecular structure and excited-state properties in these systems is highly desirable to advance the rational design of Ir(III)-based materials with pre-defined photophysical behavior.

The current contribution highlights the role of pyrene and anthracene groups in controlling the photophysical properties of cyclometalated iridium(III) complexes, with a view to their potential applications. To gain deeper insight into the structure–excited-state properties relationships, these systems have been generally classified into three categories: (i) charge-neutral Ir(III) complexes including pyrene and anthracene motifs, (ii) cationic bis-cyclometalated iridium(III) complexes bearing pyrene-functionalized ligands, and (iii) cationic mono- and bis-cyclometalated iridium(III) complexes bearing anthracene-functionalized ligands.

## 2. Neutral Ir(III) Complexes Including Pyrene and Anthracene Motifs

**Scheme 1:**
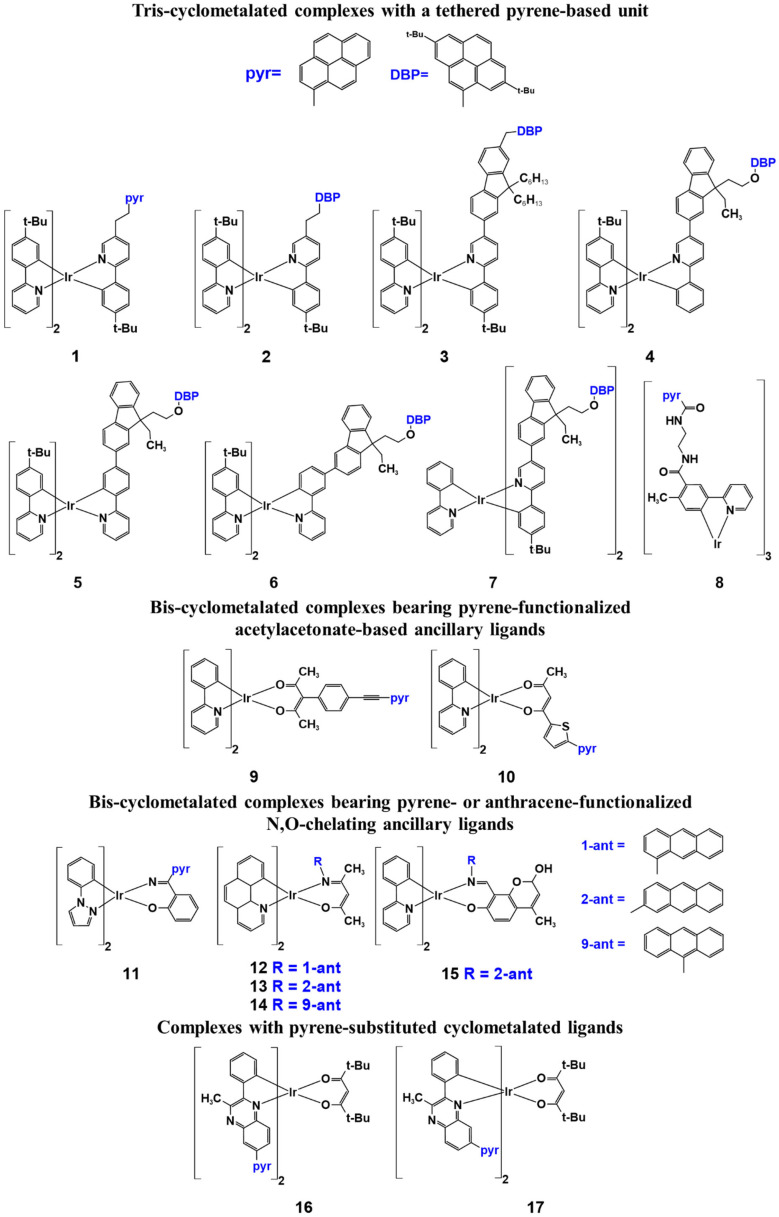

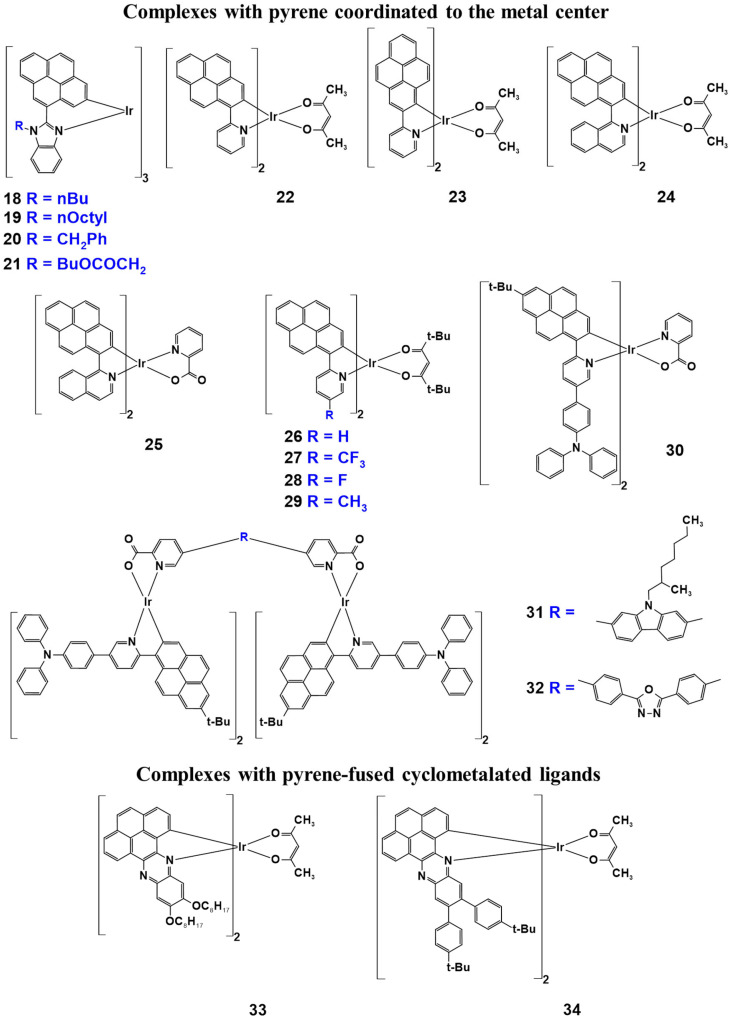
Neutral Ir(III) complexes including pyrene and anthracene motifs.

The molecular structures of Ir(III) complexes belonging to this class, which have been reported in the literature and investigated for their photophysical properties, are shown in Scheme 1. Regarding the types of structural modifications, this class comprises tris-cyclometalated complexes with tethered pyrene-based units, bis-cyclometalated complexes bearing pyrene-functionalized acetylacetonate-based ancillary ligands, bis-cyclometalated complexes bearing pyrene- or anthracene-functionalized β-ketoiminato-based ancillary ligands, complexes with pyrene-substituted cyclometalated ligands, complexes with pyrene coordinated to the metal center, and complexes with pyrene-fused cyclometalated ligands.

Among transition metal complexes, conventional tris-cyclometalated iridium(III) systems exhibit exceptionally high phosphorescence quantum yields, approaching the theoretical limit (Φ = 0.8–1.0). However, their weak visible-light absorptivity and short triplet radiative lifetimes, typically 1–5 µs, frequently preclude their effective use as photosensitizers [101,102]. A major contribution to understanding the role of pyrene units in modulating the photophysical properties of tris-cyclometalated iridium(III) complexes was provided by Zhao and Ma [19,21,103,104], who conducted a comprehensive investigation of complexes **1**–**7** in comparison with their parent chromophores lacking tethered pyrene units (**1a**–**7a**). In this well-designed series, the energy gap between the triplet excited states localized on the {Ir(ppy)_3_}- and pyrene-based units, and consequently the photophysical behavior and capacity to undergo intra- and intermolecular energy transfer processes, were tuned by varying the spatial separation between the {Ir(ppy)_3_} and pyrene moieties, attaching the pyrene–fluorene unit to different parts of the cyclometalated ligand (ppy = 2-phenylpyridine), and varying the number of pyrene–fluorene units. The UV–Vis spectra of the bichromophoric complexes **1**–**7** show well-resolved vibronic structures of the pyrene, indicating that the {Ir(ppy)_3_}-based chromophore is electronically decoupled from the appended pyrene-based group in the ground state. The π-conjugated fluorenyl linker was incorporated to enhance the visible-light absorptivity of the sensitizers [21,104]. The emission band shapes of **1**–**7** were found to be nearly identical to those for appropriate unsubstituted model chromophores **1a**–**7a**. However, the attachment of the pyrene-based motif (2,7-di-tert-butylpyrene (DBP) or pyrene) was found to markedly reduce phosphorescence quantum yields (Φ_PL_), as demonstrated in Table 1. The most pronounced reduction in Φ_PL_ was evidenced for complexes **1**, **2**, and **6**, for which the phosphorescence quantum yields were diminished to 2–7%. In contrast, the phosphorescence quantum yields of complexes **3**, **4,** and **7** decreased by approximately 30%, yet they remained relatively high at around 0.60.

For all bichromophoric systems **1**–**7**, time-resolved emission measurements revealed two dramatically distinct decay components, with nanosecond and millisecond lifetimes, respectively. The ultra-long lifetimes, spanning from 0.68 ms for **1** to 3.9 ms for **6**, were assigned to the establishment of an energetically imbalanced reversible intramolecular triplet energy transfer. The excitation of the pyrene-based chromophore via intramolecular energy transfer was confirmed using transient absorption spectroscopy [21,103]. Remarkably, such prolonged excited-state lifetimes are exceedingly rare, even among transition metal complexes renowned for their so-called “energy reservoir” behavior.

In contrast to related bichromophoric Ru(II)−pyrene systems, which exhibit nearly isoenergetic ^3^MLCT and ^3^IL_pyr_ states, the Ir(III) complexes **1**–**7** are characterized by a substantially enlarged ^3^MLCT–^3^IL_pyr_ energy gap [19]. This energetic separation promotes forward energy transfer from the {Ir(ppy)_3_} core to the pyrene chromophore, while the back energy transfer is significantly hindered (k_f_ > k_b_). As shown in Table 2, the forward and backward energy transfer rates are also influenced by the spatial separation between {Ir(ppy)_3_}- and pyrene-based units and the position of DBP or pyrene. The more strongly hindered back-energy transfer in complex **2** compared to **1** was rationalized by the reduced molecular mobility of DBP relative to pyrene, arising from steric hindrance imposed by the two bulky tert-butyl (t-Bu) groups in DBP.

In line with their ultra-long triplet lifetimes, complexes **1**–**7** were found to be promising photosensitizers. The singlet oxygen generation efficiencies of complexes **4**–**7** were evaluated using the direct method, with tetraphenylporphyrin (TPP, Φ_Δ_ = 0.7) as the reference, and were found to be excellent. All four bichromophoric systems exhibit significantly enhanced Φ_Δ_ values, surpassing those of the model chromophores and the widely used sensitizer TPP [19].

For complexes **1**, **2**, and **4**, TTA UC studies were performed both in solution and in polyurethane (PU) thin films. In all experiments, the UV-emissive DBP chromophore was used as the annihilator. Notably, the development of photosensitizers capable of efficient TTA upconversion in nonfluid and nonvolatile media is highly desirable in view of potential applications. Such studies are, however, considerably more challenging than those in solution because of reduced molecular diffusion, resulting in substantially lower upconversion efficiencies in solid matrices relative to fluid systems [104].

**Figure 1:**
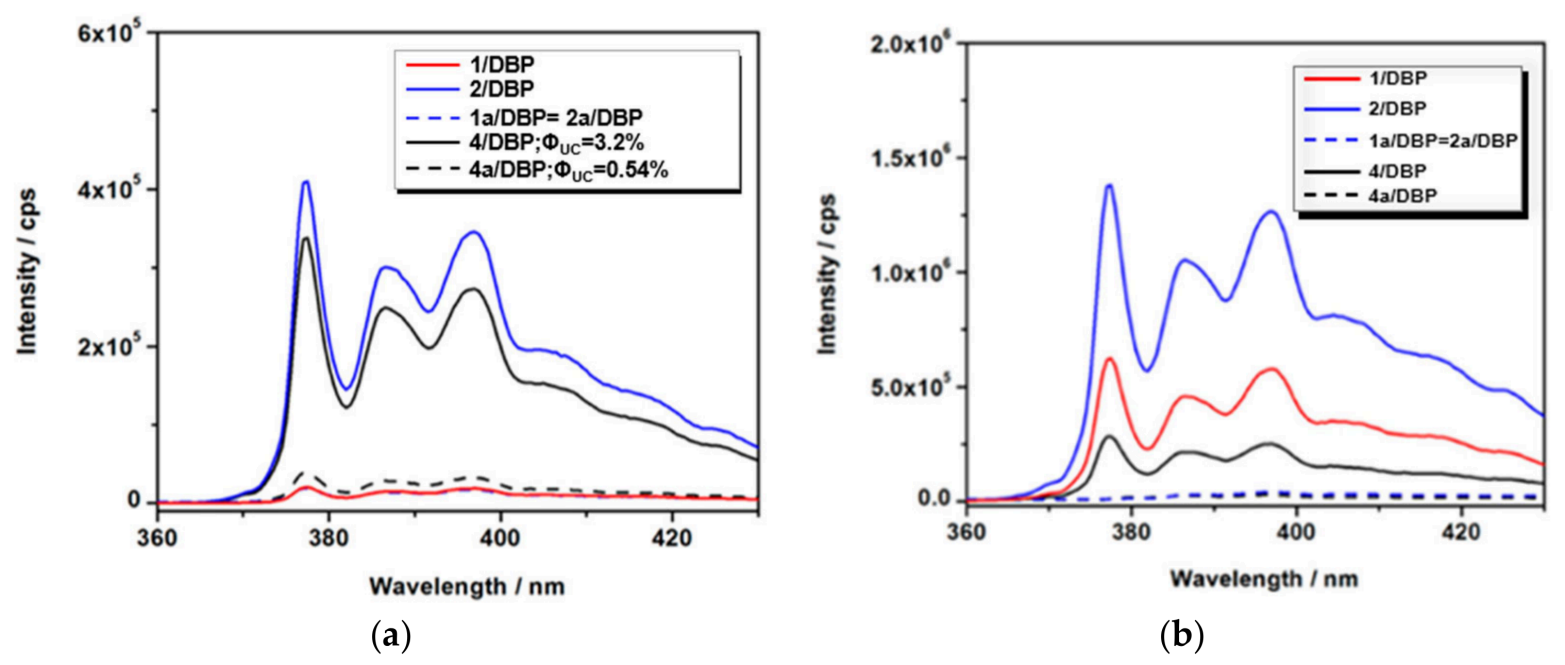
Upconversion emission intensity of deaerated toluene solutions of DBP mixed with appropriate photosensitizers at 298 K (**a**); upconversion emission intensity of PU films doped with appropriate sensitizers and DBP (**b**). Adapted with permission from [104] © 2016 American Chemical Society.

As reported in [104], measurements in toluene revealed markedly enhanced upconversion efficiencies for **2** and **4**, which were 19- and 7-fold higher, respectively, than the corresponding model chromophore (Figure 1). In contrast, the TTA efficiency of **1** was comparable to that of the model compound and significantly lower than those of **2** and **4**.

In line with the solution-phase results, **2** showed the highest upconversion efficiency in PU thin films. In contrast to toluene, however, **1**-sensitized films exhibited stronger TTA emission than those containing **4**, highlighting the importance of the shorter separation between the {Ir(ppy)_3_} and pyrene moieties for efficient solid-state TTA upconversion [104]. Enhanced photosensitizing performance in solution-phase TTA UC using DBP as the annihilator was also demonstrated for bichromophoric compounds **3** [21], **4**, and **5** [103].

Unlike complexes **1**–**7**, a prolonged excited-state lifetime was not confirmed for **8**, even though the energy gap between the triplet energy level localized on pyrene (2.13 eV) and that associated with the {Ir(tpy)_3_} unit (2.45 eV; tpy = 2-(4-tolyl)pyridine) was comparable to those of complexes **1** and **2** [24].

The relevant photoluminescence data for complex **8** and other neutral Ir(III) complexes incorporating pyrene and anthracene motifs are summarized in Table 3.

Acetylacetonate-based ancillary ligands generally do not contribute to the frontier molecular orbitals and exert only a marginal impact on the photophysical properties of bis-cyclometalated Ir(III) complexes [115]. This generalization, however, does not apply to acetylacetonate ligands functionalized with polyaromatic chromophores [10,105]. In complex **9**, the introduction of 1-ethynylpyrene into the ancillary acac-Ph ligand was found to induce substantial quenching of the ^3^MLCT emission relative to [Ir(ppy)_2_(acacPhI)] (ppy = 2-phenylpyridine), by factors of approximately 75 in THF and 250 in MeCN. Upon excitation at 280 nm, residual pyrene-centered fluorescence was observed, with maxima at 390 and 420 nm. For complex **10**, only fluorescence was detected in solution at room temperature upon irradiation into the low-energy absorption band [10]. Femtosecond transient absorption (fs-TA) studies of **9** revealed that incorporation of the 1-ethynylpyrene group introduced an additional deactivation pathway. Following excitation at 360 nm, the populated S_2_ state of pyrene undergoes two competing processes: intersystem crossing to the ^3^IL_pyrene_ state and the energy redistribution to the ^3^MLCT state. The population of the ^3^MLCT state is immediately followed by triplet-triplet energy transfer to the lower-lying ^3^IL_pyrene_ state. When excited at 420 nm, intended to selectively populate the ^1^MLCT state, deactivation proceeds predominantly via the ^1^MLCT ⟶ ^3^MLCT ⟶ ^3^IL_pyr_ pathway. The lowest triplet state of **9** is therefore localized on the pyrene chromophore, with ground state recovery occurring within 40 μs in THF and 20 μs in acetonitrile, as determined by nanosecond absorption spectroscopy. Although the intermolecular TTET was not investigated, the long-lived triplet excited state suggests that **9** can be regarded as a promising photosensitizer candidate [10].

Among the series of bis-cyclometalated complexes bearing pyrene- or anthracene-functionalized N,O-chelating ancillary ligands (**11**–**15**) [15,106,107], an extended triplet excited-state lifetime was confirmed only for complex **11**, which incorporates a pyrene moiety appended to a salicylimine-based ancillary ligand. Notably, structured phosphorescence with biexponential decay lifetimes of 18.0 and 104 μs was detected when complex **11** was dispersed in poly(methylmethacrylate) (PMMA) films. This biexponential decay behavior was attributed to dual ^3^MLCT and ^3^IL_pyr_ emission. In contrast, in solution at room temperature, complex **11** was found to be non-emissive, which was ascribed to a dominant nonradiative decay pathway arising from distortions of the six-membered O^∩^N chelate ring out of the equatorial plane. Transient absorption spectra of **11** in PMMA confirmed an excited-state absorption characteristic of ^3^IL_pyr_, which decays to the ground state within 101 μs [15].

Introduction of the pyrene motif into the 2-methyl-3-phenylquinoxaline cyclometalated ligands of complexes **16** and **17** was found to exert only a marginal impact on their photophysical characteristics in solution. Relative to the parent chromophore, both complexes show only a modest bathochromic shift (from 660 nm for the model chromophore to 673 nm for **16** and 665 nm for **17**), accompanied by a slight increase in excited-state lifetimes upon pyrene incorporation (from 1.11 μs to 1.13 μs for **16** and 1.18 μs for **17**) [108].

Prolonged excited-state lifetimes were also reported for complex **23** [110]**,** a member of the series bearing pyrene coordinated to the Ir(III) ion (complexes **18**–**32**) [28,109,110,111,112]. Although these lifetimes (11.6–125 µs) do not reach the extreme values observed for complexes **1**–**7**, they remain exceptional among pyrene-cyclometalated Ir(III) systems, with lifetimes falling in the range 0.32–5.1 µs (Table 3). Notably, this enhancement is observed even when compared with its structural isomer **22**, highlighting that even subtle variations in ligand structure can induce dramatic changes in photophysical features [110]. It was suggested that unfavorable steric interactions in **22** promote nonradiative deactivation through an additional vibrational mode [108]. Remarkably, in contrast to **22**, the excited-state lifetimes of **23** were found to be significantly impacted by solvent polarity, being almost five times longer in non-polar toluene than in polar acetonitrile.

Ir(III) complexes incorporating a pyrene-fused diazaacene core (**33** and **34**) were found to be a promising alternative for the development of efficient deep-red emitters. These complexes exhibit emission maxima beyond 700 nm, sub-microsecond excited-state lifetimes, and photoluminescence quantum yields of approximately 14% (Table 3). These highly π-extended and rigid ligands were proven to effectively suppress nonradiative decay pathways in Ir(III) systems, thereby enhancing their suitability for applications in organic light-emitting diodes (OLEDs) [113,114].

## 3. Cationic Bis-Cyclometalated Iridium(III) Complexes Bearing Pyrene-Functionalized Ligands

**Scheme 2:**
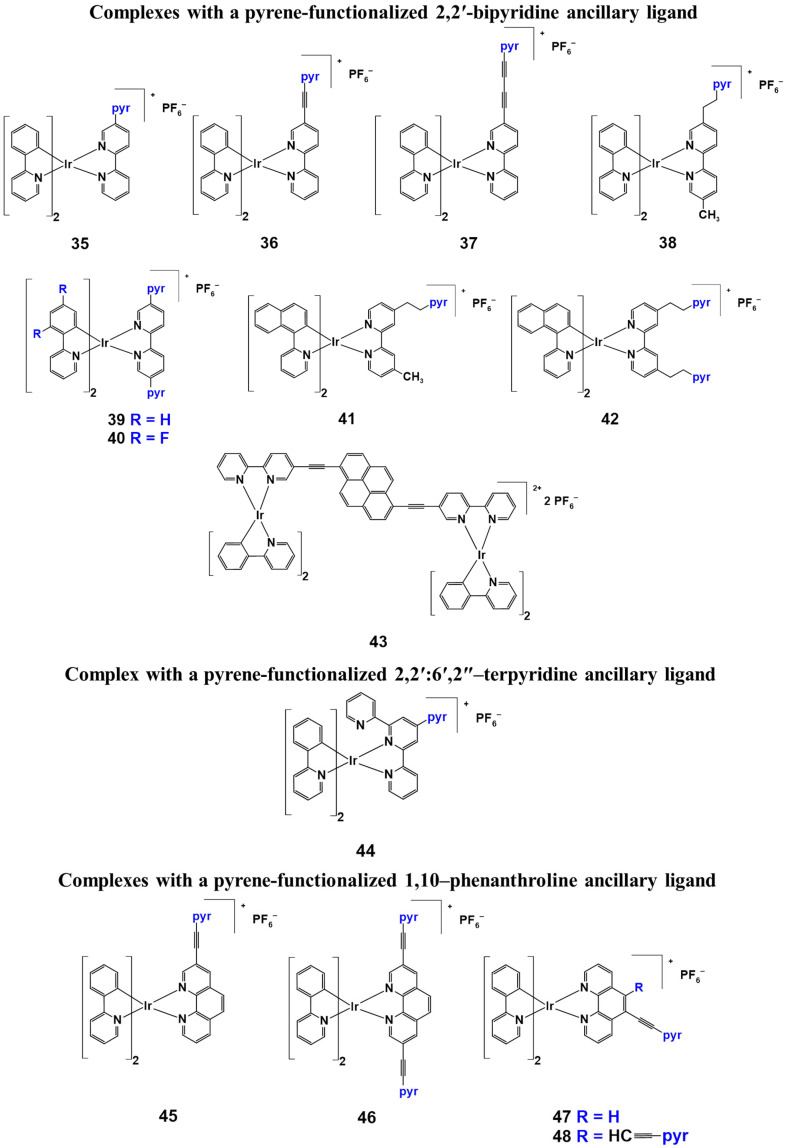

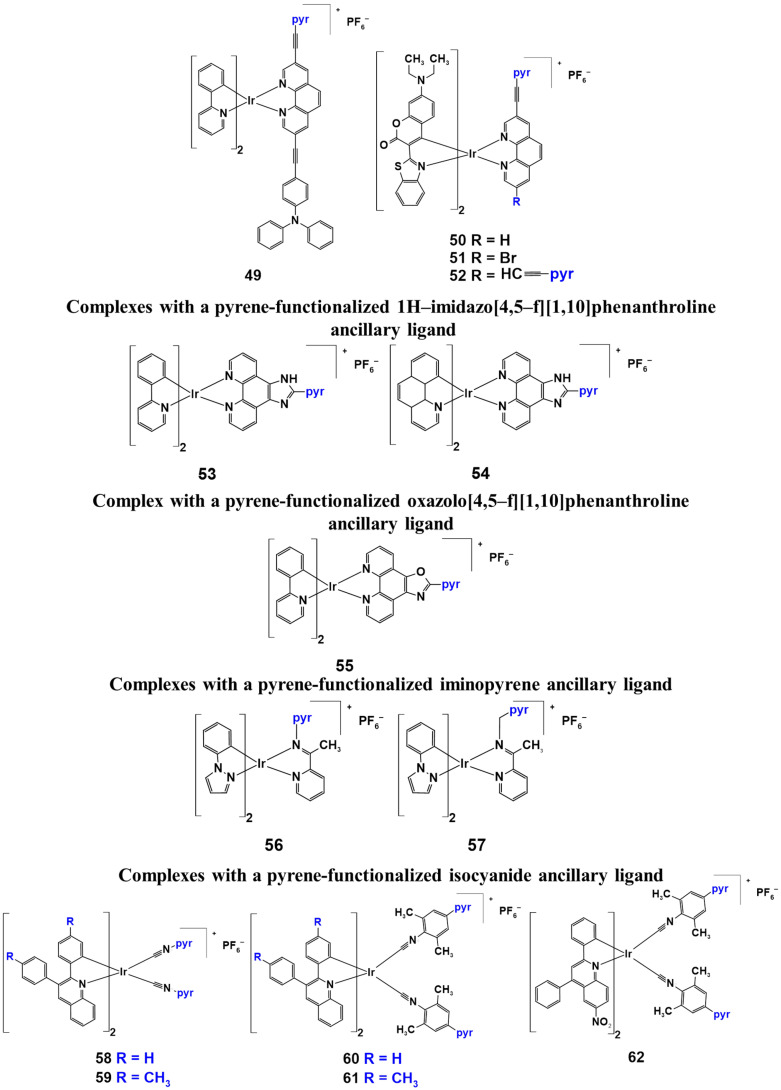

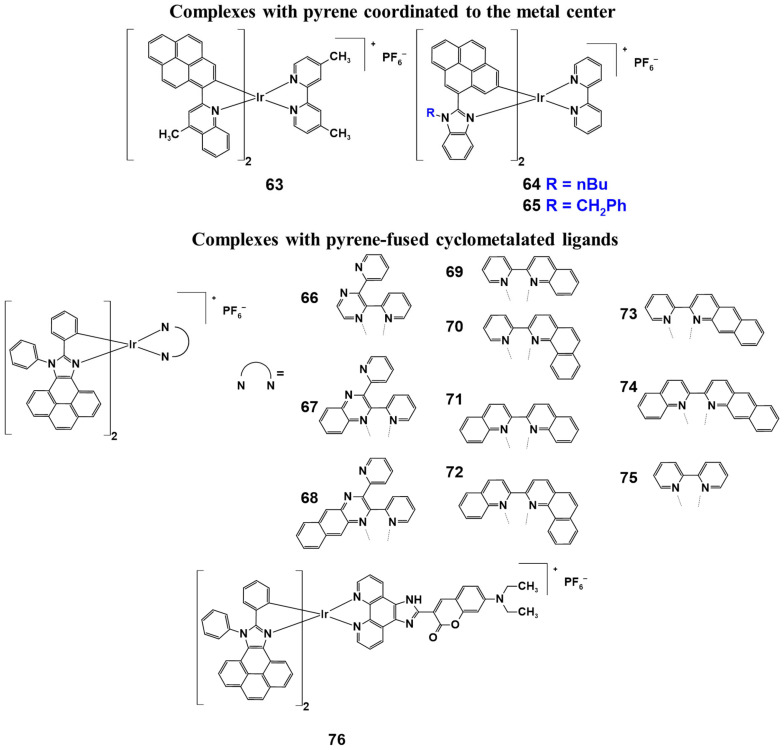
Cationic bis-cyclometalated iridium(III) complexes bearing pyrene-functionalized ligands.

The cationic bis-cyclometalated iridium(III) complexes with pyrene-functionalized ligands reported in the literature are illustrated in Scheme 2. Their photophysical properties are summarized in Table 4 and provided in more detail in Table S3.

Consistent with the larger π electron delocalization degree due to the introduction of π-conjugated pyrene, most of these systems exhibit red-shifted and enhanced absorption in the visible region relative to the corresponding unsubstituted complexes [43,109,116,117]. Furthermore, the visible-light absorption capacity increases (i) upon replacing a single bond with a triple bond, and subsequently with a conjugated double–triple bond linkage between the pyrenyl and Ir(III)-based chromophores (**35**, **36** and **37**); (ii) upon increasing the number of pyrenyl units (**45** and **46**); and (iii) upon the introduction of additional electron-donating groups (such as triphenylamine in **49**) and the use of strongly absorbing cyclometalating ligands (such as 3-(2-benzothiazolyl)-7-(diethylamino)coumarin in **50**–**52**) [43,49,50,51]. The clear vibronic fine structure characteristic of pyrene between 300 and 350 nm, observed in the UV–Vis spectra of **38**–**42**, **45**–**48**, **50**–**52, 56** and **57**, is indicative of weak electronic coupling between the Ir(III)-based and organic chromophores [14,15,16,49,51,118,119].

Apart from complex **54**, whose photophysical properties were not investigated, and complexes **44**, **56**, **58**, and **59**, which are non-emissive in solution at room temperature, all other bis-cyclometalated iridium(III) complexes incorporating pyrene-functionalized ligands exhibit photoluminescence (Table 4). In general, the functionalization of ancillary ligands with pyrene or the incorporation of cyclometalated pyrene generally increases nonradiative decay rates (k_nr_), resulting in reduced emission quantum yields compared to the corresponding unsubstituted complexes (Tables S3 and S4).

**Table 4:** Photoluminescence properties of cationic bis-cyclometalated Ir(III) complexes bearing pyrene-functionalized ligands.

Compound	Medium	λ_exc_ [nm]	λ_PL_ [nm]	τ [µs]	τ_TA_ [µs]	φ_PL_	Ref.
**35**	MeCN	420	648, ~710	–	53.3	–	[43]
**36**			671, ~740	–	60.1	–	
**37**			679, ~750	–	60.5	–	
**38**	CHCl_3_	350	~400, 425, 560	0.0018 (97%) 0.520 (3%)	–	–	[118]
**39**	DCM	~450	668, 740 (sh)	0.004	–	0.003	[14]
	77 K	–	663, 725	~1	–	–	
**40**	DCM	–	665, 740 (sh)	0.007	–	0.006	
	77 K	–	663, 725	~1	–	–	
**41**	MeCN	465	590, 625	225	225	0.095	[16]
	77 K	413	~600, 615, 650, 660	–	–	–	
	MeCN	–	590, 625	225	–	0.095	[119]
	BuCN 77 K	413	~600, 615, 650, 660	–	–	–	
**42**	MeCN	–	590, 625	480	–	0.096	
	BuCN 77 K	413	~600, 615, 650, 660	–	–	–	
**43**	MeCN	420	683, ~750	–	56.7	–	[43]
**45**	MeCN		667, ~740	–	92.4	–	
	DCM	440	672, 747	136.1	157.2	0.01	[49]
	77 K		~670, 740	–	–	–	
**46**	DCM	480	682, 757	73.1	85.8	0.013	
	77 K		~670, 760	–	–	–	
**47**	DCM	440	678, 751	213.1	367.7	0.001	
	77 K		~660, 725	–	–	–	
**48**	DCM	440	600	1.3	247.1	0.005	
			738	90.8			
	77 K		~580, 760	–	–	–	
**49**	DCM	482	677, ~740	46.3	53.3	0.027	[50]
	EtOH/MeOH (4:1, *v*/*v*) 77 K	482	~670, 750	122.8	–	–	
**50**	DCM	440	680, ~760 (sh)	172.8	195.5	0.006	[51]
	DCM 77 K	–	~670, 750	586.4	–	–	
**51**	DCM	440	685, ~770 (sh)	77.5	72.8	0.009	
	DCM 77 K	–	~670, 750	472.7	–	–	
**52**	DCM	440	690, ~770 (sh)	67.2	68.5	0.007	
	DCM 77 K	–	~685	261.6	–	–	
**53**	MeCN	375	574	–	5.08	0.0011	[25]
**55**	MeCN	–	548	0.60	–	–	[116]
	2-MeTHF 77 K	–	658, 721	–	–	–	
	Toluene	–	–	–	28.5	0.68	
	Toluene/10% DCM	436	541, 664	–	–	–	
	THF		585, 665, 730	–	–	–	
	DCM		577, 664	–	–	–	
	Acetone		548, 659	–	–	–	
	MeCN		548	0.6	–	–	
**56**	PMMA	–	631	0.329 2.4	0.378 2.9	0.004	[15]
**57**	DCM	–	615	2.7	3.0	0.014	
	PMMA	–	610	2.0 28.1	0.378 2.9	0.028	
**58**	DCM/toluene (1:3) 77 K	280	626, 642, 679, 696, 716, 764, 824	–	–	–	[29]
	MeCN	–	–	–	22	–	
**59**	DCM/toluene (1:3) 77 K	450	626, 643, 658, 680, 697, 716, 763, 824	–	–	–	
	MeCN	–	–	–	31	–	
**60**	DCM/toluene (1:3) 77 K	330	615, 678, 750 sh	–	–	–	
	MeCN		427	0.01	22	<0.001	
**61**	DCM/toluene (1:3) 77 K		616, 670	–	–	–	
	MeCN		439	0.013	18	<0.001	
**62**	DCM/toluene (1:3) 77 K		613, 676, 736 sh	–	–	–	
	MeCN		403	0.011	2.4	<0.001	
**63**	DCM	410	704, 771 sh	–	–	–	[117]
	THF		–	3.11	–	0.0014	
**64**	CHCl_3_	–	435	0.0027	13.3	–	[109]
**65**	CHCl_3_	–	407	0.0026	3.9	–	
**66**	DCM	–	651	0.185	–	0.036	[120]
	MeCN	–	655	0.12	0.119 0.110 0.108	0.014	
	THF	–	649	0.17	–	0.026	
	Toluene (10% DCM)	–	654	0.12	–	0.019	
**67**	DCM	–	710	0.07	–	0.0046	
	MeCN	–	714	0.03	0.055 0.056	0.0014	
	THF	–	705	0.04	–	0.0033	
	Toluene (10% DCM)	–	712	0.05	–	0.0029	
**68**	DCM	–	810	0.38	–	–	
	MeCN	–	803	0.32	0.392 0.405 0.380	–	
	THF	–	808	0.24	–	–	
	Toluene (10% DCM)	–	791	0.28	–	–	
**69**	DCM	–	625	0.94	–	0.13	[121]
	MeCN	–	628	0.68	0.65 0.65 0.67	0.06	
	THF	–	624	0.84	–	0.13	
	Toluene (10% DCM)	–	622	0.47	–	0.08	
**70**	DCM	–	593	0.15	–	0.017	
	MeCN	–	600	–	15.3 15.0 16.1	0.0089	
	THF	–	600	–	–	0.0097	
	Toluene (10% DCM)	–	598	–	–	0.0096	
**71**	DCM	–	657	0.93	–	0.12	
	MeCN	–	663	0.45	0.56 0.46 0.47	0.049	
	THF	–	657	0.58	–	0.057	
	Toluene (10% DCM)	–	672	0.20	–	0.020	
**72**	DCM	–	645	1.34	–	0.085	
	MeCN	–	–	–	13.1 14.9 13.9	–	
	THF	–	645	1.29	–	0.041	
	Toluene (10% DCM)	–	645	0.36	–	0.030	
**73**	DCM	–	740	2.21	–	0.0049	
	MeCN	–	735	2.11	2.80 2.68 2.82	0.0028	
	THF	–	739	1.95	–	0.0039	
	Toluene (10% DCM)	–	731	2.01	–	0.0045	
**74**	DCM	–	766	0.86	–	0.0039	
	MeCN	–	766	0.71	0.86 0.83 0.87	0.0024	
	THF	–	765	0.87	–	0.0032	
	Toluene (10% DCM)	–	765	0.37	–	0.0025	
**75**	Toluene	404	569		56.1	0.076	[122]
	EtOH/MeOH (4:1 *v*/*v*) 77 K		~560, 575, 625, 680	–	–	–	
**76**	Toluene	460	607		73.9	0.034	
	EtOH/MeOH (4:1 *v*/*v*) 77 K		~600, 650, 720	–	–	–	

**Figure 2:**
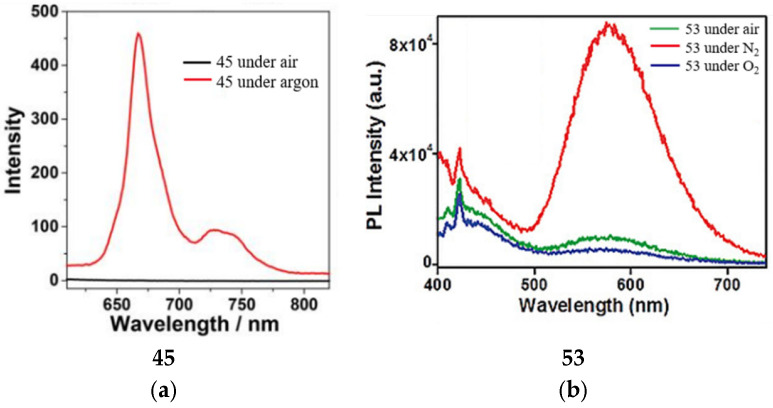

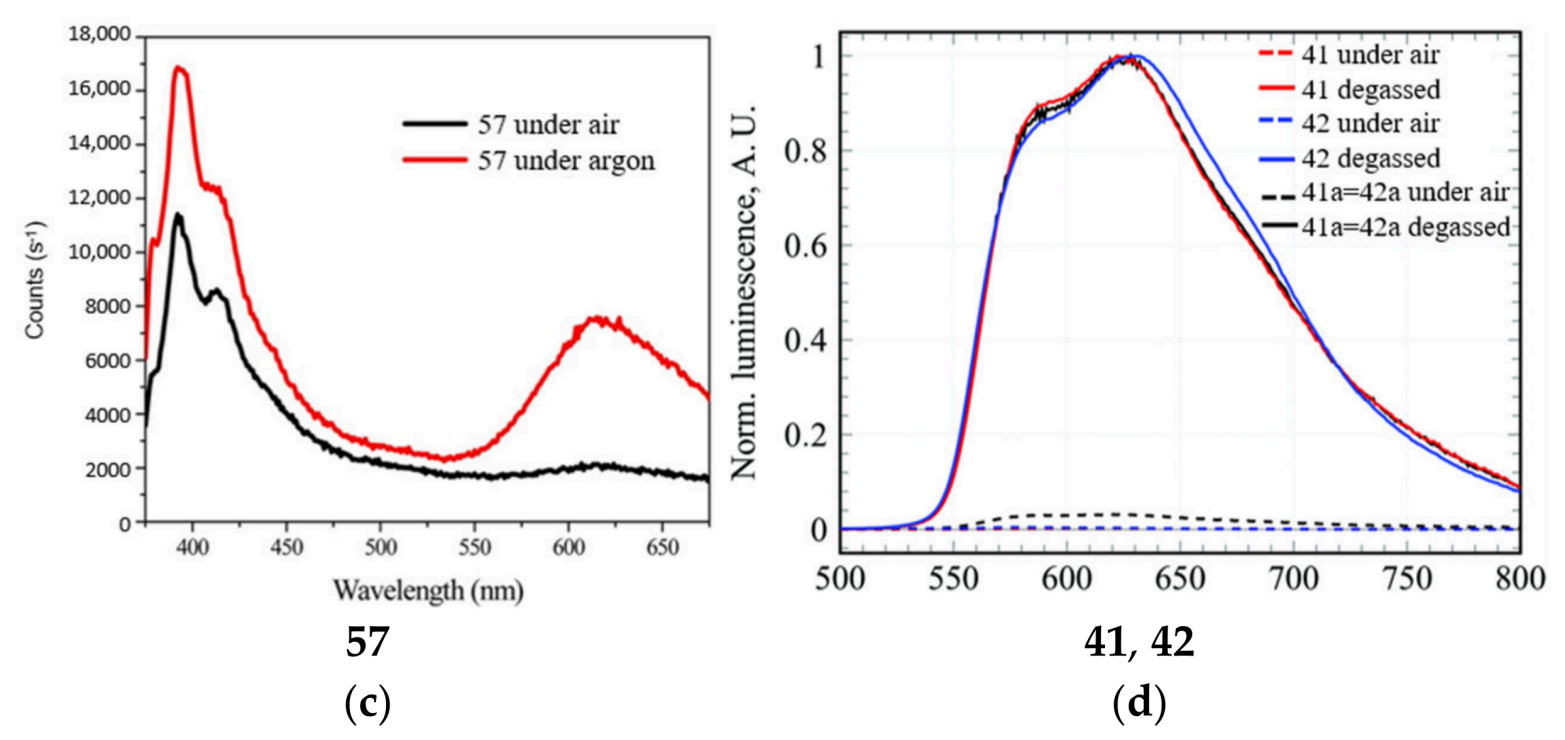
Emission spectral profiles of representative complexes belonging to this class (**a**–**d**). Adapted with permission from [43]. Copyright © 2023 Wiley-VCH GmbH, [25]; ©2020 Wiley-VCH Verlag GmbH & Co. KGaA, Weinheim, [15] © 2014 American Chemical Society, [119] © The Royal Society of Chemistry 2016.

Considering their photophysical behavior, bis-cyclometalated iridium(III) complexes bearing pyrene-functionalized ancillary ligands constitute a highly diverse group. Complexes **35**–**37**, **39, 40**, **43**, **45**–**47**, and **49**–**51** exhibit room-temperature emission in solution that originates from the ^3^IL_pyr_ excited state. The emission appears in the red region, shows pronounced vibrational progression under an inert atmosphere (Ar or N_2_), and is efficiently quenched upon exposure to air [14,43,49]. In contrast, complexes **41**, **42**, **53**, and **55** emit at higher energies relative to the aforementioned compounds. Their emission bands, attributed to ^3^MLLCT or mixed ^3^MLLCT/^3^IL character, are featureless or show very weak vibronic structure (Figure 2) [16,119]. In turn, **48** and **57** represent rare examples of dual-emissive Ir(III) systems. In the case of complex **48**, radiative decay occurs from both ^3^MLCT (600 nm) and ^3^IL_pyr_ (738 nm) excited states, while complexes **38** and **57** display emission from ^1^IL_pyr_ (~400 nm) and ^3^MLCT (560–600 nm) [15,118]. Moreover, in poly(methyl methacrylate) (PMMA) films, dual emission ^3^MLCT and ^3^IL_pyr_ was also confirmed for complexes **56** and **57** [15].

The bis-cyclometalated Ir(III) complexes with 2,6-dimethyl-4-(1-pyrenyl)phenyl isocyanide ligands display only weak residual fluorescence from the ^1^IL_pyr_ state in the 400–440 nm range (**60**–**62**). Notably, their analogues containing 1-pyrenyl isocyanides (**55** and **56**) show no luminescence at room temperature, highlighting the crucial role of steric effects in controlling the photobehavior of these systems [29]. A pyrene-localized fluorescent emissive state was also confirmed for complexes in which pyrene is coordinated to the metal center (**64, 65**), with the exception of **63**, which shows phosphorescence originating from a ^3^IL_pyr_-dominated excited state [109,117]. The observation of fluorescence instead of phosphorescence is indicative of an ISC efficiency lower than 100% for this class of complexes [109]. In turn, iridium(III) complexes with pyreno [4,5-d]imidazole C^∩^N ligands (**66**–**76**) emit from ^3^MLCT/^3^LLCT or ^3^IL_N-N_ excited states, depending on the degree of π-conjugation and structural modifications of the N^∩^N ligands [120,121,122].

The emissive states of **41** and **42** in solution at room temperature demonstrate extremely long-lived excited-state lifetimes of 225 and 480 μs, respectively. The prolonged emission lifetimes of these systems, relative to the model chromophores (Tables S3 and S4) arise from reversible electronic energy transfer ^3^MLCT ⟶ ^3^IL_pyr_, as demonstrated by transient absorption studies [16,119]. Table 5 presents the rate constants estimated for forward (k_f_) and backward (k_b_) electronic energy transfer processes in the excited-state equilibrium ^3^MLCT ↔ ^3^IL_pyr_ along with the energy gap between the ^3^MLCT and ^3^IL_pyr_ excited states for complexes **41** and **42**.

Using transient absorption spectroscopy, intramolecular energy transfers and the nature of the lowest triplet state were also investigated for complexes **35**–**37**, **41**, **43**, **45**–**53, 55**–**62**, and **64**–**76**. Triplet lifetimes determined using ns-TA spectroscopy (nanosecond transient absorption) are gathered in Table 4. For all investigated bis-cyclometalated iridium(III) complexes bearing pyrene-functionalized ancillary ligands, the lowest triplet state was found to be localized on the pyrene chromophore [15,16,25,29,43,49,50,51,109,116,120,121,122]. Noteworthy, for complex **44**, which has sufficiently large two-photon absorption cross-sections in the NIR region, the energy transfer from the singlet excited state of the 4-(pyren-1-yl)-terpy (pyr-terpy) ligand to the Ir(III) moiety and subsequently back again to the triplet excited state of pyr-terpy (so-called ‘‘ping-pong’’ mechanism) was firstly confirmed upon two-photon excitation. For this complex, the lowest singlet excited state of pyr-terpy (3.31 eV) lies significantly higher in energy than the Ir(III)-centered ^1^MLCT state (2.64 eV), whereas the lowest triplet state of pyr-terpy (2.05 eV) is markedly lower in energy than ^3^MLCT (2.50 eV). Consequently, the fluorescence of pyr-terpy is efficiently quenched via singlet–singlet energy transfer to the ^1^MLCT state, which promptly undergoes intersystem crossing (ISC) to the ^3^MLCT. Subsequently, the ^3^MLCT state undergoes triplet-triplet energy transfer to the ^3^IL_pyr_, completing the ‘‘ping-pong’’ mechanism [123].

**Figure 3:**
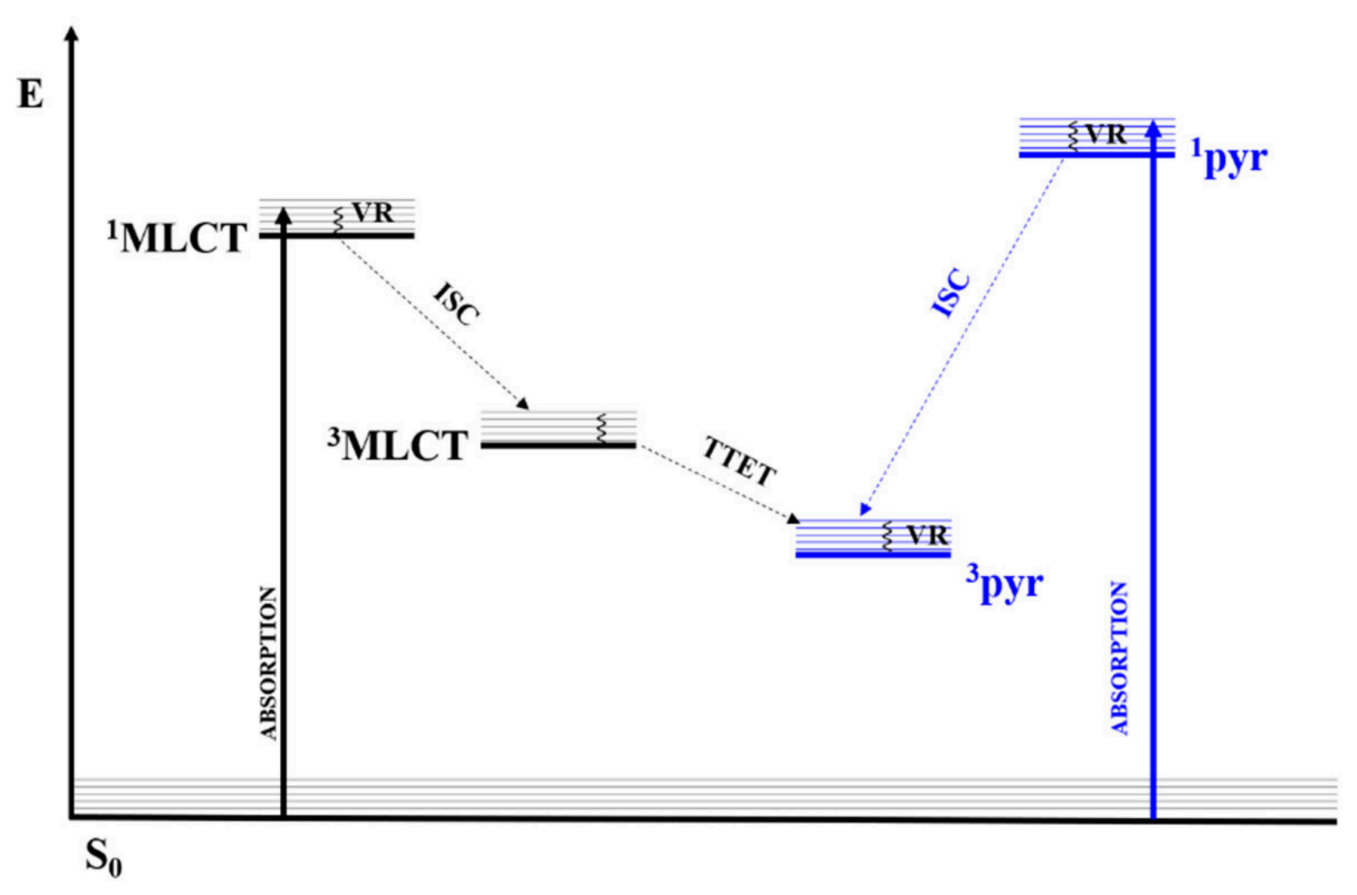
Two possible pathways of population of ^3^IL_pyr_, proposed for complexes bearing pyrene-substituted isocyanide ligands [29].

The studies of bis-cyclometalated Ir(III) complexes bearing pyrene-substituted isocyanide ligands revealed that the population of ^3^IL_pyrene_ can be achieved by two pathways, ^1^IL_pyrene_ ⟶ ^3^IL_pyrene_ and ^1^MLCT ⟶ ^3^MLCT ⟶ ^3^IL_pyrene_ [29], as illustrated in Figure 3.

TA (Transient Absorption) studies of iridium(III) complexes with pyreno [4,5-d]imidazole C^∩^N ligands demonstrated that the character of the lowest triplet excited states is predominantly determined by the nature of the ancillary ligands, resulting in ^3^MLCT/^3^LLCT character for **66**, **67**, **69**, and **71** and ^3^IL_N-N_ in the cases of **68**, **70**, **72**–**74**, and **76** [120,121,122]. Notably, the lifetime (73.9 μs) of the lowest triplet excited state of **76** localized on the coumarin unit is among the longest lifetimes for Ir(III) complexes of this type (Table 4).

The long-lived triplet excited states and improved visible-light absorptivity make cationic bis-cyclometalated iridium(III) complexes with pyrene-functionalized ligands promising candidates for further investigation as photosensitizers. The photosensitizing performance of **35**–**37**, **43**, and **45** was evaluated in the photoreduction of CO_2_ to CO, which is considered a highly attractive approach for mitigating environmental pollution and alleviating the global energy shortage. The catalytic process was demonstrated to proceed through the following stages: (i) photoexcitation to the singlet state upon visible-light irradiation of the Ir(III) complex; (ii) intersystem crossing and generation of the triplet state of the photosensitizer; (iii) formation of the reduced form of the Ir(III) complex upon electron transfer from 1,3-dimethyl-2-phenyl-2,3-dihydro-1H-benzo[d]imidazole; and (iv) subsequent electron transfer to the catalyst [Fe(qpy)(OH_2_)_2_]^2+^ (qpy = 2,2′:6′,2″:6″,2′′′-quaterpyridine), which drives CO_2_ photoreduction. The CO yields follow the order **35** < **45** < **37** < **36** < **43** and correlate well with the increase in the visible-light absorption capacity of the Ir(III) complexes, indicating that enhanced absorption in the visible region is another crucial photosensitizing factor alongside extended excited-state lifetimes. The highest photocatalytic activity (Table 6) was evidenced for the dinuclear Ir(III) compound **43**, which exhibits markedly increased visible-light absorptivity (ε: 57,300 M^−1^⋅cm^−1^ vs. 3720 M^−1^⋅cm^−1^) and a substantially prolonged triplet excited-state lifetime (56.7 μs vs. 0.30 μs) relative to[Ir(ppy)_2_(bpy)]^+^. The CO yield obtained with this dinuclear complex exceeded that of [Ir(ppy)_2_(bpy)]^+^ by more than a factor of 54 [43].

Energy transfer from photosensitizers **44**, **50**–**53**, **64**, and **65** to the ground-state molecular oxygen (^3^O_2_), resulting in the formation of highly reactive singlet oxygen (^1^O_2_), was investigated by an indirect method with the employment of 1,3-diphenylisobenzofuran (DPBF) and 1,5-dihydroxynaphthalene (1,5-DHN) as a ^1^O_2_ scavengers and [Ru(bipy)_3_]Cl_2_, methylene blue, or meso-tetraphenylporphyrin as standards. As demonstrated in Table 7, all investigated compounds show highly efficient ^1^O_2_ generation capabilities, with the highest singlet oxygen quantum yield observed for **44** [25,51,109,123].

The generation of ^1^O_2_ represents the type II photosensitization pathway in photodynamic therapy (PDT), offering several advantages over conventional chemotherapy, including higher efficacy, reduced invasiveness, fewer adverse side effects, and improved selectivity. Upon two-photon excitation, which utilizes a low-energy NIR laser as a light source and minimizes side effects due to reduced interaction between NIR light and the tissue, the singlet oxygen generation efficiency was evaluated only for complex **44**. In addition, its highly efficient intracellular ^1^O_2_ generation capability was demonstrated in human cancer cell lines SKOV-3 and A549 (Table 8) [123].

The photosensitizing capability of **45**–**52**, **75**, and **76** was investigated in the triplet–triplet annihilation upconversion (TTA UC), where the photoexcited triplet-state energy of the PS is transferred to the acceptor/annihilator through intermolecular Dexter-type triplet-triplet energy transfer. Subsequent collision between two triplet-excited annihilator molecules, ^3^(annihilator)*, induces bimolecular triplet-triplet annihilation (TTA), in which one molecule relaxes to the ground state while the other is promoted to a higher-energy singlet excited state, ^1^(annihilator)*. This singlet state then undergoes radiative decay, giving rise to delayed fluorescence. TTA UC processes, which enable the conversion of low-energy photons into higher–energy emission, have attracted considerable attention for applications in renewable energy technologies, including solar energy conversion devices that rely on efficient light harvesting, photoexcitation, and charge separation. To determine the photosensitizing capability of these systems, 9,10-diphenylanthracene (DPA) was selected as a triplet annihilator due to its high fluorescence quantum yield and its appropriate T_1_ energy level (1.77 eV) relative to the triplet energy level of the pyrene-functionalized Ir(III) photosensitizer. The upconversion data, including the Stern–Volmer quenching constants, bimolecular quenching constants, upconversion quantum yields and overall upconversion capability (η) are given in Table 9. These data demonstrate that complexes **45**, **46**, **49**, and **50** are among the most efficient PSs for TTA UC.

## 4. Cationic Mono- and Bis-Cyclometalated Iridium(III) Complexes Bearing Anthracene-Functionalized Ligands

**Scheme 3:**
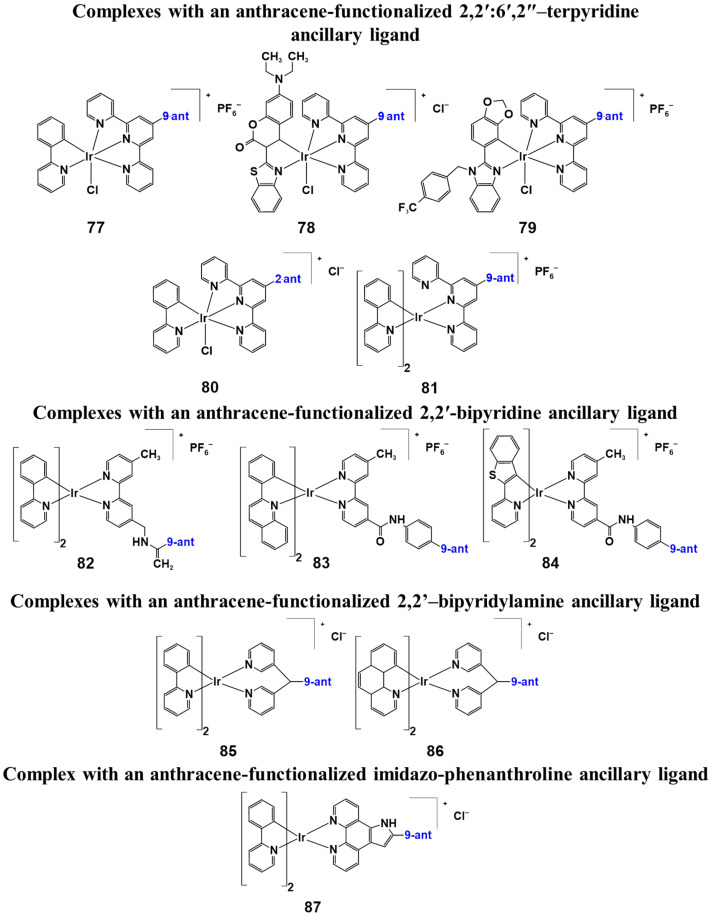

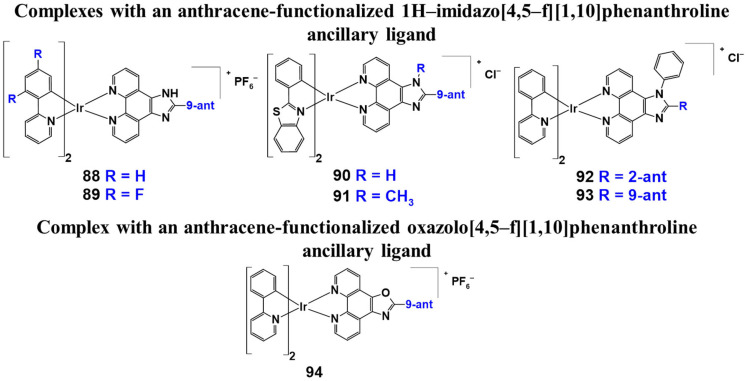
Cationic mono- and bis-cyclometalated iridium(III) complexes bearing anthracene-functionalized ligands.

The role of the anthracene group in controlling photophysical behavior has been investigated for mono- and bis-cyclometalated iridium(III) complexes given in Scheme 3. The absorption and emission properties of these systems are summarized in Table S5. Mono-cyclometalated Ir(III) compounds with anthryl-substituted ancillary ligands remain largely unexplored compared with bis-cyclometalated iridium(III) complexes bearing anthryl-functionalized diimines. To date, all reported cationic mono-cyclometalated iridium(III) complexes feature 2,2′:6′,2″-terpyridine derivatives, namely, 4′-(9-anthryl)-2,2′:6′,2″-terpyridine and 4-(2-anthryl)-2,2′:6′,2″-terpyridine, coordinated to the Ir(III) center in a tridentate fashion (**77**–**80**) [124,125,126,127].

The UV–Vis spectra of complexes **81**, **83**–**86**, **88**–**91**, and **93** display distinct anthracene vibrational features in the 340–390 nm region, followed by weak metal-to-ligand charge-transfer (MLCT) absorption extending into the 450–500 nm range [128,129,130,131,132]. This spectral profile indicates relatively weak electronic coupling between the Ir(III)-centered chromophore and the anthracene unit. In contrast, compounds **77**, **79**, **80**, **82**, **87**, **92**, and **94** exhibit broad absorption in the 350–500 nm range, most likely attributable to mixed ^1^MLCT and ^1^IL/^1^ILCT transitions, rather than ^1^MLCT/^1^LLCT character [31,116,124,125,126,132,133]. By analogy with cationic bis-cyclometalated iridium(III) complexes bearing pyrene-functionalized ligands, the incorporation of strongly absorbing cyclometalating ligands (such as coumarin-based frameworks in complex **78**) results in the appearance of an intense absorption ^1^IL/^1^MLCT band in the 400–550 nm range [125].

The photoluminescence properties of cationic mono- and bis-cyclometalated iridium(III) complexes bearing anthracene-functionalized ligands are gathered in Table 10. In general, mono- and bis-cyclometalated iridium(III) complexes bearing anthracene-functionalized ligands are weakly emissive. They typically display broad, featureless emission profiles consistent with ^3^MLLCT-type emission observed for related model chromophores, however, in contrast to the unsubstituted systems, they are characterized by low photoluminescence quantum yields (Table S6). This behavior arises from efficient ^3^MLCT excited-state quenching by the anthracene unit, which occurs via intramolecular energy transfer from the ^3^MLCT state to the substantially lower-lying triplet excited state localized on the organic chromophore (~1.85 eV). The excited-state lifetimes in solution at room temperature fall within the range of 0.037–3.41 µs. Complexes **88**–**91** were reported to be non-luminescent in solution at room temperature, while **85**–**87** represent rare examples that exhibit fluorescence–phosphorescence emission [31,130,131]. In turn, the green fluorescence of **78** may most likely attributed to emission from the coumarin-based cyclometalating ligand [125,134].

Using TA spectroscopy, the lowest-energy anthryl-based triplet state was confirmed for **94**. The TA lifetime of this complex was approximately one order of magnitude longer than its emission lifetime, indicating that the emitting and TA-detected states have different origins [116].

A remarkable feature observed for complexes **83**, **84**, **88**, **89**, and **90** is a gradual decrease in the intensity of the absorption bands characteristic of the anthracene unit upon prolonged irradiation. This behavior is rationalized by photooxidation of the anthracene moiety by ^1^O_2_, generated during photoexcitation of the Ir(III) complexes via intermolecular triplet-triplet energy transfer from the anthracene triplet state (^3^Ant) to molecular oxygen (^3^O_2_) [129,131,134].

**Scheme 4:**
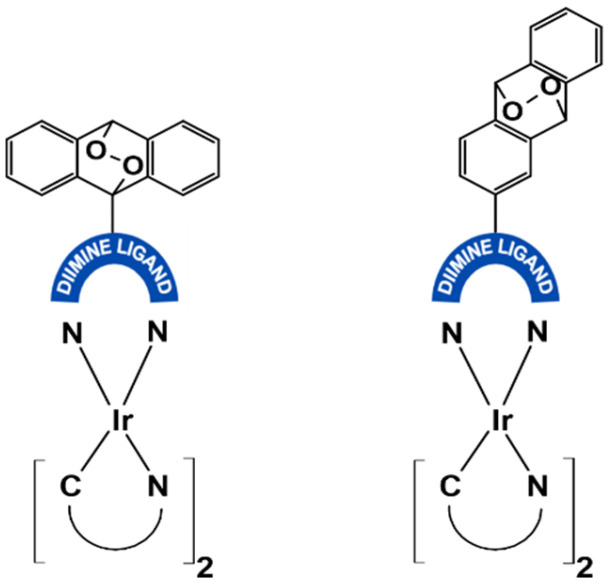
Schematic representation of endoperoxide forms of anthracene units in bis-cyclometalated iridium(III) complexes bearing diimine ligand.

Upon reaction with ^1^O_2_, anthracene forms an endoperoxide, in which the 9- and 10- carbon atoms are bridged by two oxygen atoms (Scheme 4). The formation of Ir(III) complexes bearing anthracene endoperoxide moieties was further confirmed by electrospray ionization mass spectrometry as well as by ^1^H and ^13^C NMR spectroscopy [129,131,132,134].

The authors of [132,134] demonstrated that the photooxidation activity of Ir(III) complexes bearing imidazo [4,5-f][1,10]phenanthroline ligands can be effectively tuned by the dihedral angle between the imidazo-phenanthroline framework and the anthracene moiety. This modulation can be achieved by employing different anthryl linking modes (2-anthryl versus 9-anthryl) and by introducing steric substituents at the N–H position of the imidazole ring. In general, an increase in the dihedral angle leads to a weakening of the photooxidation activity of Ir(III) complexes incorporating anthryl-functionalized imidazo [4,5-f][1,10]phenanthroline ancillary ligands.

**Figure 4:**
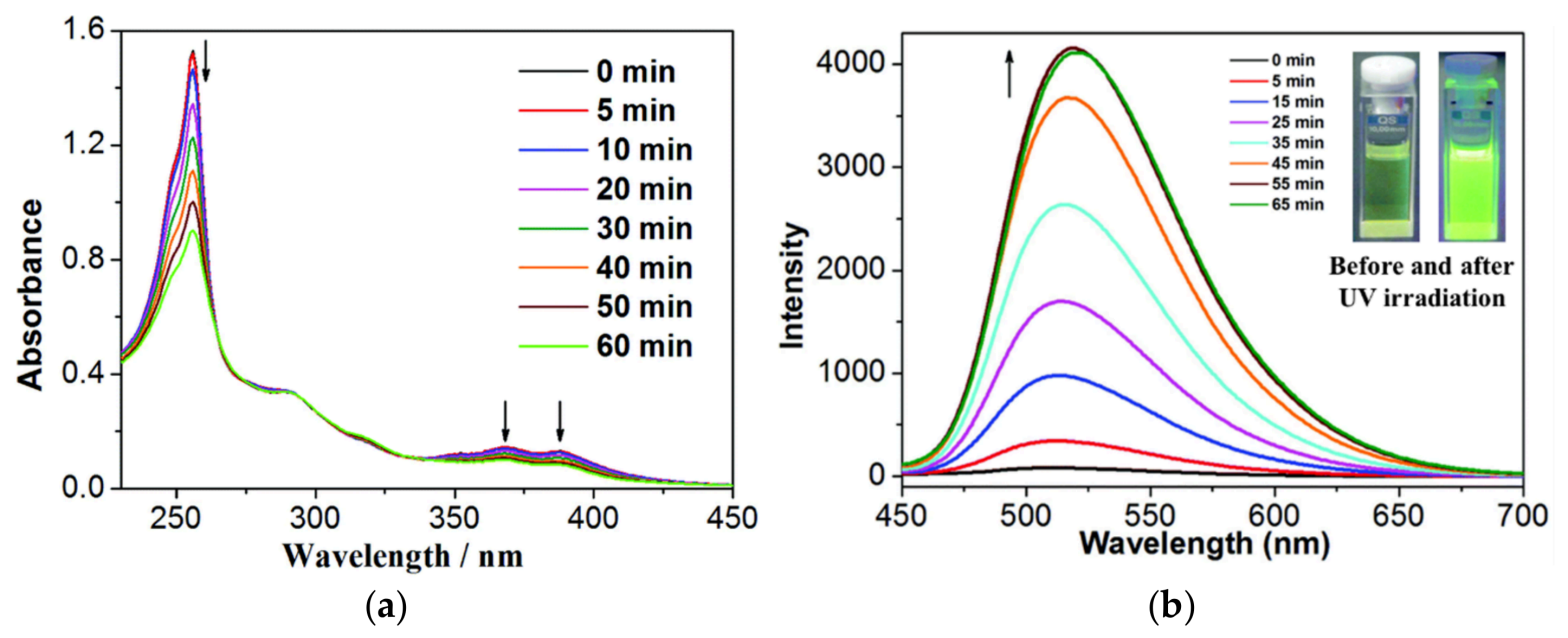
(**a**) UV–Vis absorption spectral changes of **89** [131] in CH_2_Cl_2_ upon irradiation with 365 nm light; (**b**) luminescence spectral changes of **89** [131] in CH_2_Cl_2_ upon irradiation with 365 nm light (c = 10^−4^ M, λ_ex_ = 380 nm). Adapted with permission from [131] Copyright © The Royal Society of Chemistry 2017.

**Scheme 5:**
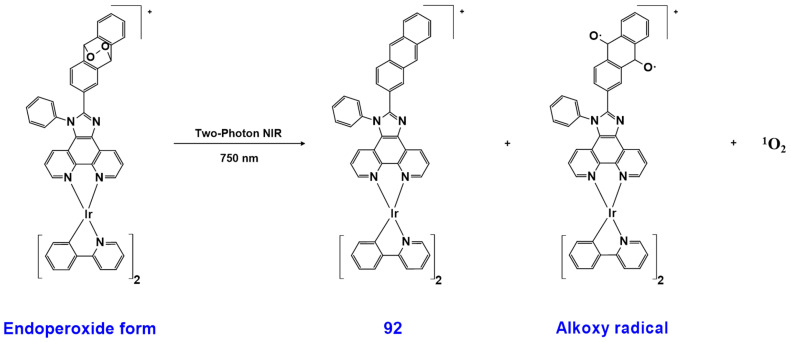
Reaction upon two-photon irradiation of the endoperoxide form of **92**.

Notably, the capture of singlet oxygen by Ir(III) complexes **83**, **84**, **88**, **89**, **90**, **92**, and **93** was also evidenced to be responsible for the pronounced enhancement of their emission intensity, consistent with the inhibition of energy transfer from the Ir(III) fragment to the anthracene moiety (Figure 4b) [129,131,132,134]. This feature enables anthracene-based Ir(III) complexes to serve as efficient luminescent probes for imaging ^1^O_2_, including in biological systems. The suitability of **83** for detecting intracellular ^1^O_2_ was demonstrated using laser-scanning confocal microscopy and flow cytometry [129]. Furthermore, the endoperoxide form of **92** was identified as a very promising mitochondria-localized prodrug for synergistic photodynamic therapy and photoactivated chemotherapy. Upon two-photon near-infrared (NIR) irradiation, it was shown to release a highly cytotoxic Ir(III) complex **92**, ^1^O_2_, and an alkoxy radical under hypoxic conditions, as illustrated in Scheme 5 [132].

Energy transfer from anthryl-functionalized Ir(III) complexes to ground-state molecular oxygen (^3^O_2_) and the formation of highly reactive singlet oxygen (^1^O_2_) were investigated for complexes **78**, **79**, and **87** using an indirect method based on monitoring changes in the absorbance of 1,3-diphenylisobenzofuran (DPBF). As demonstrated in Table 11, the substantial singlet oxygen quantum yield value via the type II mechanism was confirmed for complex **79** [31,125,126]. A high ability to generate singlet oxygen from molecular oxygen was also confirmed for the endoperoxide form of **92**, determined under normoxic conditions using the nonfluorescent dichlorodihydrofluorescein diacetate probe [132].

Several anthryl-functionalized Ir(III) complexes were found to generate reactive oxygen species (ROS) also via an electron-transfer pathway to biomolecules, consistent with a type I photochemical mechanism. Among the most promising biological targets is nicotinamide adenine dinucleotide (NADH), an essential coenzyme involved in cellular metabolism, ATP production, and the maintenance of redox homeostasis. Since cancer cells require elevated levels of NADH compared with normal cells due to their rapid growth, NADH photooxidation, mediated by metal-based photocatalysts, has emerged as an effective anticancer strategy, referred to as photocatalytic cancer therapy [125]. The photocatalytic potential for the conversion of NADH to NAD+ was investigated for complexes **78**, **79**, and **87** [31,125,126], and the relevant parameters concerning NADH photooxidation activity of these systems are given in Table 12. Complex **78** represents the most effective photocatalyst for NADH oxidation among anthryl-functionalized Ir(III) complexes.

Additionally, complexes **78**, **79**, and **87** were found to be able to generate other radicals (•OH, O_2_^•−^) through a type I mechanism, as demonstrated by methylene blue [125], 3,3′,5,5′-tetramethylbenzidine [31], and non-fluorescent hydroxyphenyl fluorescein [126] assays.

The photochemotherapeutic effect was studied for complexes **78**, **79**, **80**, **85**, **86**, **87**, and **92** [31,125,126,127,130,132]. Their anticancer activity in the dark and upon exposure to visible light was summarized in Table 13.

Finally, the beneficial impact of appended anthryl groups was also demonstrated in terms of the photocatalytic activity of Ir(III) complexes for the conversion of carbon dioxide. Mono-cyclometalated Ir(III) complex with 4′-(9-anthryl)-2,2′:6′,2″-terpyridine (**77**) has been shown to photoreduce CO_2_ to CO more efficiently relative to the unsubstituted model complex (**77a**), as illustrated in Table 14. The enhanced photocatalytic performance of **77** was assigned to the steric effect of the 9-anthryl substituent and the low-lying anthracene triplet state [124].

## 5. Conclusions

In summary, owing to their structural versatility, large spin–orbit coupling constants, and good chemical and photostability, cyclometalated iridium(III) metal complexes may be considered promising platforms for the development of efficient photosensitizers for applications in photocatalysis, TTA UC, PDT, and PACT. In general, further improvement in their photosensitizing performance requires increasing visible-light absorptivity and extending triplet-state lifetimes. We have clearly demonstrated the adaptability of the bichromophoric strategy in the design of iridium(III) complexes as PSs. The review addresses a discernible gap in the current literature by providing a detailed discussion of the role of pyrene and anthracene groups in controlling the photophysical properties of cyclometalated iridium(III) complexes, particularly in the context of their potential applications as photosensitizing agents.

Incorporation of pyrene-based motifs into tris-cyclometalated iridium(III) systems provides a powerful strategy for achieving exceptionally long triplet excited-state lifetimes, extending into the millisecond regime. These unusually prolonged lifetimes arise from an energetically imbalanced reversible intramolecular triplet energy-transfer process. Such compounds remain exceedingly rare among transition metal complexes and highlight the unique photophysical potential of this design approach. Notably, the visible-light absorptivity of these sensitizers may be substantially enhanced by the introduction of strongly absorbing linkers between the Ir(III)-based core and pyrene chromophore. Consistent with their ultra-long triplet lifetimes, these systems exhibit remarkable photosensitizing performance, efficiently generating singlet oxygen and enabling enhanced triplet-triplet annihilation upconversion. Although the introduction of pyrene-based motifs generally leads to a noticeable decrease in Φ_PL_, rational molecular design allows the development of tris-cyclometalated iridium(III) complexes that retain high emissive efficiencies, with Φ_PL_ values approaching 0.60.

Related photoinduced processes, including excitation of the pyrene-based chromophore via intramolecular energy transfer and formation of reversible electronic energy transfer ^3^MLCT → ^3^IL_pyr_, were also demonstrated for cationic bis-cyclometalated iridium(III) complexes with pyrene-functionalized ligands. Although triplet excited-state lifetimes in these systems do not reach the extreme values observed for tris-cyclometalated iridium(III), they remain exceptionally long among Ir(III) coordination compounds. Numerous examples from this family have proven to be efficient PSs for photoreduction of CO_2_ to CO, ^1^O_2_ generation, and TTA UC processes. Particularly noteworthy is the complex [Ir(Phpy)_2_(pyr-terpy-κ^2^N)]PF_6_, in which pyrene-functionalization leads to an enhanced absorption cross-section in the NIR region, enabling its two-photon excitation. Remarkably, this compound exhibits an exceptional singlet oxygen quantum yield of 0.98, and its PS activity has been validated in human cancer cell lines SKOV-3 and A549.

Relative to bichromophoric Ir(III)−pyrene systems, mono- and bis-cyclometalated Ir(III) compounds with anthryl-substituted ancillary ligands are characterized by a substantially enlarged ^3^MLCT–^3^IL_pyr_ energy gap, which promotes an efficient ^3^MLCT excited-state quenching through intramolecular energy transfer to the considerably lower-lying triplet excited state localized on the anthracene unit. For anthryl-functionalized Ir(III) complexes, photosensitizing abilities have been demonstrated in the photoreduction of CO_2_ to CO, the generation of singlet oxygen via a type II mechanism, and the formation of reactive oxygen species (ROS) via a type I photochemical mechanism. A particularly distinctive feature of some of these systems is their capacity to capture singlet oxygen, leading to the formation of Ir(III) complexes bearing anthracene endoperoxide moieties. These endoperoxide-containing systems have been identified as extremely promising mitochondria-targeted prodrugs capable of enabling synergistic photodynamic therapy combined with photoactivated chemotherapy.

Overall, the structure–activity relationships highlighted in this review underline both the potential and current limitations of pyrene- and anthracene-functionalized cyclometalated Ir(III) compounds as PSs, as well as providing useful guidelines for the rational development of next-generation Ir(III)-based systems for applications in photocatalysis, TTA UC, PDT, and PACT.

## Figures and Tables

**Table 1:** Photoluminescence (PL) properties with upconversion data of complexes **1**–**7** and their parent chromophores **1a**–**7a** recorded in toluene solution at room temperature under deoxygenated conditions (see also Tables S1 and S2 for other photophysical properties of **1**–**7** and **1a**–**7a**).

Compound	λ_PL_ [nm]	Lifetimes		Quantum Yield Φ_PL_	Upconversion Quantum Yield Φ_UC_ [%]	Ref.
		τ_1_ [µs]	τ_2_ [µs]			
**1**	510	0.0020	680	0.03		[104]
**2**	511	0.0024	2800	0.02	2.6	
**3**	565	0.001	2200	0.66	2.4	[21]
**4**	562 563 *	0.012 0.0105 *	1000 1000 *	0.59 0.60 *	3.2	[103] [19] *
**5**	553 * 552 *	0.0087 0.0079 *	2000 2000 *	0.19 0.31 *	4.1	[103] [19] *
**6**	520	0.0074	3900	0.07		[19]
**7**	564	0.0040	1200	0.64		
**1a** = **2a**	510	1.3		0.97		[104]
**3a**	565	2.2		0.92	0.45	[21]
**4a**	561 560 *	2.3 2.2 *		0.92 0.92 *	0.54	[103] [19] *
**5a**	550	3.8		0.78 0.81 *	0.26	[103]
**6a**	517	1.4		0.97		[19]
**7a**	563	2.9		0.90		

*—show which results come from which work—they are marked with asterisks.

**Table 2:** The calculated parameters of intermolecular triplet-triplet energy transfer processes in complexes **1**–**7** with the singlet oxygen generation efficiencies of complexes **4**–**7**.

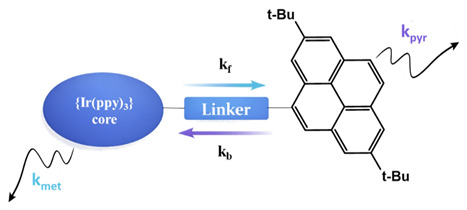								
Compound	k_met_ [10^6^ s^−1^] ^a^	k_f_ [s^−1^] ^b^	k_b_ [s^−1^] ^c^	k_pyr_ [s^−1^] ^d^	K ^e^	ΔE [eV] ^f^	Φ_Δ_	Ref.
**1**	0.76	5 × 10^8^	3 × 10^4^	1430	17,000	0.43	–	[104]
**2**	0.76	4 × 10^8^	4 × 10^3^	350	114,000	0.39	–	
**3**	0.45	9.1 × 10^8^	8.1 × 10^5^	128	1100	0.17	–	[21]
**4**	0.45	0.95 × 10^8^	1.48 × 10^5^	350	640	0.17	~0.8	[19]
**5**	0.26	1.27 × 10^8^	4.70 × 10^4^	320	2600	0.20		
**6**	0.71	1.35 × 10^8^	3.1 × 10^3^	260	44,000	0.27		
**7**	0.34	2.50 × 10^8^	4.63 × 10^5^	270	540	0.14		

**^a^ k_met_**—the decay rate of the metal-complex chromophore; **^b^ k_f_**—the forward energy transfer rate constant; **^c^ k_b_**—the backenergy transfer rate constant; **^d^ k_pyr_**—the decay rate of the pyrene chromophore; **^e^ K**—the equilibrium constant of the reversible triplet energy transfer from the Ir complex to the attached pyrene; **^f^ ΔE**—D–A energy gap determined using the lowest triplet energy levels of the Ir chromophores and DBP, estimated from their emission maxima at 77 K. The values of **k_met_**, **k_pyr_**, and **K** were estimated using the following equations: $\frac{1}{\tau_{1}}\approx k_{f}+k_{b};\;\frac{1}{\tau_{2}}=\frac{1}{1+K}kmet+\frac{K}{1+K}k_{pyr};\;K=\frac{k_{f}}{k_{b}}\;$ [19].

**Table 3:** Photoluminescence (PL) properties of complexes **8**–**30**.

Compound	Medium	λ_exc_ [nm]	λ_PL_ [nm]	τ [µs]	Φ_PL_	Ref.
**8**	DMSO	366	388, 409, 505	1.0	<0.01	[24]
**9**	THF	430	390, 420, ~520	–	–	[10]
	MeCN	460	–	0.0021	–	
**10**	DCM	410	563	–	<0.01	[105]
**11**	PMMA	–	615	18.0 and 104	0.15	[15]
**12**	Chlorobenzene	485	~570	–	–	[106]
**13**			~570	–	–	
**14**			~570	–	–	
**15**	DCM	–	471, 440	0.00281 and 0.00715, 0.00188 and 0.00698	0.011	[107]
**16**	2-MeTHF	–	673	1.13	0.136	[108]
**17**	2-MeTHF	–	665	1.18	0.189	
**18**	CH_3_Cl	–	425	0.0027	–	[109]
**19**		–	429	0.0028	–	
**20**		–	425	0.0025	–	
**21**		–	446	0.0030	–	
**22**	DCM	400	680, 734	2.7	0.0056	[110]
	Toluene		684, 734	2.5	0.0013	
	MeCN		680, 734	2.5	0.0088	
	EPA		680, 738	3.6	–	
	EPA 77 K		679, 745	5.1	–	
**23**	DCM	430	626, 683	37.0	0.063	
	Toluene		626, 680	52.9	0.021	
	MeCN		623, 685	11.6	0.060	
	EPA		623, 685	67.1	–	
	EPA 77 K		622, 636, 676, 692	125.0	–	
**24**	DCM	–	~705	–	–	[111]
**26**	DCM	436	681	0.42	0.0031	[28]
	Hexane	–	680	–	–	
	Toluene	–	683	–	–	
	MeCN	–	676	–	–	
**27**	DCM	436	692	0.80	0.0033	
	Hexane	–	692	–	–	
	Toluene	–	695	–	–	
	MeCN	–	689	–	–	
**28**	DCM	436	680	0.32	0.0026	
	Hexane	–	682	–	–	
	Toluene	–	682	–	–	
	MeCN	–	676	–	–	
**29**	DCM	436	679	0.47	0.0024	
	Hexane	–	679	–	–	
	Toluene	–	682	–	–	
	MeCN	–	677	–	–	
**30**	DCM	440	698, 792 sh	1.04	0.0225	[112]
	DCM 77 K		693, 760 sh	4.82	–	
**31**	DCM		699, 792 sh	1.41	0.007	
	DCM 77 K		702, 773 sh	5.05	–	
**32**	DCM		700, 792 sh	1.42	0.0122	
	DCM 77 K		704, 778 sh	4.67	–	
**33**	DCM	450	732	0.60	0.13	[113]
	2-MeTHF 77 K		706, 776	–	–	
	Toluene		~715	–	–	
	THF		~715	–	–	
	CHCl_3_		~750	–	–	
**34**	DCM	600	789	0.22	0.14	[114]

**Table 5:** Reversible electronic energy transfers in complexes **41** and **42**.

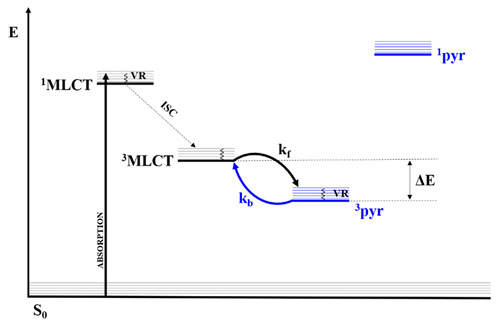					
Compound	ΔE [eV]	k_f_ [s^−1^]	k_b_ [s^−1^]	k_eq_	Ref.
**41**	0.08	19.3 × 10^7^	0.7 × 10^7^	27.8	[16]
**42**	0.09	55 × 10^7^	0.9 × 10^7^	61	[119]

**Table 6:** Summary of the factors that can affect the sensitizing ability of **35**–**37**, **43**, and **45**.

Compound	φ_CO_ [µmol]	ε [M^−1^·cm^−1^]	τ [µs]	Ref.
**35**	23.5	9600	53.3	[43]
**36**	60.1	33,000	60.1	
**37**	45.3	41,500	60.5	
**43**	91.5	57,300	56.7	
**45**	33.5	30,200	92.4	

**Table 7:** Schematic presentation of singlet oxygen trapping by DPBF and 1,5-DHN, along with the singlet oxygen quantum yields for compounds **44**, **50**–**53**, **64**, and **65**.

Singlet oxygen trapping by DPBF and 1,5–DHN 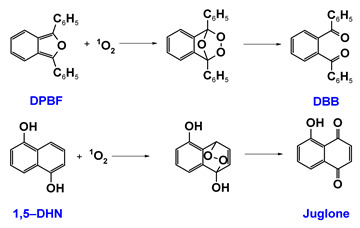			
Compound	Φ_Δ_	Experimental Details	Ref.
**44**	0.98	MeOH, λ_exc_ = 440 nm, DPBF [Ru(bipy)_3_]Cl_2_ as standard	[123]
**50**	0.815	DCM, λ_exc_ = 410 nm, DPBF [Ru(bipy)_3_]Cl_2_ as standard	[51]
**51**	0.845		
**52**	0.823		
**53**	0.73	Benzene, λ_exc_ = 355 nm, DPBF TPP as standard	[25]
**64**	0.86 (97.8 *)	DCM, λ_exc_ = 420 nm, 1,5-DHN TPP as standard	[109]
**65**	0.80 (99.2 *)		

* Yield of Juglone after reaction for 60 min [109]; DBB—1,2-dibenzoylbenzene.

**Table 8:** IC_50_ values (µM) of complex **44**. PI represents the phototoxicity index (PI = Dark IC_50_/Light IC_50_).

Dark	SKOV–3	6.31 ± 0.48	A549	3.62 ± 0.27
Light		0.0027 ± 0.0002		0.0034 ± 0.0004
PI		2337		1065

**Table 9:** Upconversion data for complexes **45**–**52**, **75**, and **76**.

Compound	Medium	τ_TA_ [µs] ^a^	K_SV_ × 10^3^ [M^−1^] ^b^	k_q_ × 10^9^ [M^−1^ × s^−1^] ^c^	Φ_UC_ [%] ^d^	ε × 10^4^ [M^−1^ × cm^−1^]	η × 10^6^ [M^−1^ × cm^−1^] ^e^	Ref.
**45**	DCM	157.2	629.9	5.03	30.2	3.07	0.93	[49]
**46**		85.8	227.9	3.34	31.6	5.24	1.66	
**47**		367.7	847.4	4.68	20.9	2.86	0.59	
**48**		247.1	30.7	0.15	7.9	2.45	0.19	
**49**		53.3	474	9.67	28.1	5.09	1.43	[50]
**50**		195.5	388.1	2.4	27.5	13.1	3.60	[51]
**51**		72.8	133.1	1.9	18.2	12.9	2.35	
**52**		68.5	126.1	2.2	10.6	13.4	1.42	
**75**	toluene	56.1	130.6	–	–	1.91	–	[122]
**76**		73.9	146.7	–	23.7	5.15	1.22	

**^a^** Triplet lifetimes; **^b^** Stern–Volmer quenching constant; **^c^** bimolecular quenching constants; **^d^** upconversion quantum yield; **^e^** upconversion capability (η = ε × Φ_UC_).

**Table 10:** Photoluminescence properties of cationic mono- and bis-cyclometalated Ir(III) complexes bearing anthracene-functionalized ligands.

Compound	Medium	k_r_ (10^5^ s^−1^)	k_nr_ (10^5^ s^−1^)	λ_exc_ [nm]	λ_PL_ [nm]	τ [µs]	τ_TA_ [µs]	φ_PL_	Ref.
**77**	MeCN	0.25	2.69	380	553	3.41	–	0.0844	[124]
**78**	DMSO:PBS (0.5:99.5 *v*/*v*)	–	–	460	512	–	–	–	[125]
**79**	MeCN	–	–	330	545	0.0361 (3%) 0.23 (97%)	–	0.018	[126]
**80**	MeOH	–	–	730 *	565	–	–	0.017	[127]
**81**	MeCN	17,400	26,000	–	440	0.23	–	0.40	[128]
**82**	1% DMSO/H_2_O	–	–	450	~580	0.037	–	–	[133]
**83**	Carbonate buffer (0.1 M, pH = 10.5) containing Na_2_MoO_4_ (without addition of H_2_O_2_ for 83, 84 and after addition of H_2_O_2_ for 83 ^ep^, 84 ^ep^)	–	–	–	625	–	–	<0.01	[129]
**83 ^ep^**		–	–	–	625, I/I*_o_* = 20	–	–		
**84**		–	–	–	640	–	–	<0.01	
**84 ^ep^**		–	–	–	640, I/I*_o_* = 15	–	–	–	
**85**	DMSO	–	–	437	486, 545, 551	0.186	–	0.083	[130]
**86**	DMSO	–	–	432	521, 551	0.242	–	0.064	
**87**	MeCN	6.53	13.88	382	438, 572	0.049	–	0.32	[31]
**88**	MeCN	–	–	–	–	–	–	–	[131]
**88 ^ep^**	DCM	–	–	–	578	0.122, 0.223	–	0.125	
		–	–	–	+TFA: 582	0.067, 0.210	–	–	
**89**		–	–	–	–	–	–	–	
**89 ^ep^**		–	–	–	519	0.138, 0.468	–	0.372	
		–	–	–	+TFA: 528	0.271, 0.495	–	–	
**90**	DCM	–	–	397	–	–	–	–	[134]
**90 ^ep^**		–	–		~525, 565, 615	–	–	–	
**91**		–	–		–	–	–	–	
**91^ep^**		–	–		~525, 560, 615	–	–	–	
**92**	MeOH	–	–	405	592	0.365 *	–	–	[132]
**92 ^ep^**		–	–		592	0.874 **	–	–	
**93**		–	–		608	0.452 **	–	–	
**93 ^ep^**					608	0.630 **	–	–	
**94**	MeCN	0.18	25	436	590	0.390	–	0.007	[116]
	Toluene/10% DCM	–	–		588	0.390	–	0.004	
	THF	–	–		589	0.600	–	0.13	
	DCM	–	–		580	0.690	–	0.015	
	Acetone	–	–		590	0.390	–	0.008	
	2-MeTHF 77 K	–	–		555 600	4.62 3.89	–	–	
	Toluene	–	–	355	–	–	24.4 3.57, 25.0	–	

* At the maximum of the two-photon absorption cross-section; ** The lifetime was recorded under hypoxia conditions; ^ep^ endoperoxide form; I/I*_o_* –emission enhancement factors.

**Table 11:** Singlet oxygen quantum yields (Φ_∆_) along with experimental details.

Compound	Φ_Δ_	Experimental Details	Ref.
**78**	0.05	DMSO:PBS (0.5:99.5, *v*/*v*)	[125]
		525 nm	
		[Ru(bipy)_3_]Cl_2_ as standard	
**79**	0.77	MeCN	[126]
		465 nm	
		[Ru(bipy)_3_]Cl_2_ as standard	
**87**	0.26	DMSO	[31]
		Visible light irridation (400–700 nm)	
		Rose Bengal	
**92 ^ep^**	0.79	Under normoxic conditions using the nonfluorescent dichlorodihydrofluorescein diacetate probe	[132]
**93 ^ep^**	0.16	405 nm	

^ep^—endoperoxide forms.

**Table 12:** Photooxidation of NADH via singlet oxygen (^1^O_2_) generated by Ir(III)-based photocatalysts, along with experimental details and turnover number (TON) and turnover frequency (TOF) parameters for complexes **78**, **79**, and **87**.

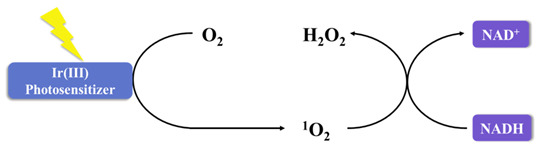				
Compound	Conditions	TON	TOF [h^−1^]	Ref.
**78**	Complex conc. (1 μM)	108	1084.0	[125]
	NADH (150 μM) in DMSO:PBS (0.5:99.5, *v*/*v*)			
	525 nm, 50.2 J·cm^−2^			
**79**	Complex conc. (5 μM)	5.76	8.64	[126]
	NADH (100 μM) in PBS:DMF (95:5)			
	465 nm, 4.6 mW·cm^−2^			
**87**	Complex conc. (5 to 50 µM)	4.54	37.82	[31]
	NADH (100 μM) in DMSO-PBS			
	400–700 nm, 10 J·cm^−2^			

**Table 13:** IC_50_ values (µM) of complexes **78**, **79**, **80**, **85**, **86**, **87**, and **92**. PI represents the phototoxicity index (PI = Dark IC_50_/Light IC_50_).

	**78** [125]							
Dark	MCF–7	0.6 ± 0.1	HeLa		7.0 ± 0.2	HEK–293		13.4 ± 1.3
Light		0.3 ± 0.1 **^a^**			1.0 ± 0.1			7.9 ± 0.4
PI		2.0			7.0			–
	**79 ^b^** [126]							
Dark	HeLa	10 ± 2	HCT116	3.5 ± 0.9	A375	2.7 ± 0.9	MRC5pd30	12 ± 2
Light		0.14 ± 0.04		0.026 ± 0.003		0.037 ± 0.005		–
PI		69		135		72		–
	**80** [127]		**85** [130]		**86** [130]	**87** [31]		
Dark	A549	12.5 ± 1.3	13.6 ± 0.08		18.9 ± 0.12	MDA–MB–231	>300	
Light		4.6 ± 1.3 **^c^** 3.3 ± 0.8 **^d^**	1.16 ± 0.06 **^e^** 0.18 ± 0.05 **^f^**		1.35 ± 0.08 **^e^** 0.40 ± 0.08 **^f^**		3.8 ± 0.34	
PI		3.8	11.7 ^e^ 75.5 ^f^		14.0 **^e^** 47.2 **^f^**		78.94	
			**92 ^g^** [132]			**92 ^h^** [132]		
Dark	A549		7.47 ± 1.92			88.1 ± 2.32		
Light			5.01 ± 1.63 ^i^			0.73 ± 1.74 ^i^		
PI			1.5			120.7		

**^a^** Irradiated in the 400–700 nm range, 5 J·cm^−2^, for 10 min; **^b^** irradiated at 420 nm by an LED area light (50 ± 3 W·cm^−2^) for 1 h; **^c^** irradiated at 405 nm using an LED area light (20 mW·cm^−2^) for 10 min; **^d^** irradiated at 730 nm using a laser light (25 mW) for 10 min; **^e^** irradiated at 405 nm using an LED lamp (6.5 J·m^2^) for 10 min; **^f^** irradiated at 470 nm by an LED lamp (15.1 J·m^2^) for 10 min; **^g^** hypoxic conditions (1% O_2_); **^h^** normoxic conditions (21% O_2_); ^i^ irradiated using a two photon near-infrared (TP-NIR) laser at 750 nm (50 mW).

**Table 14:** Photocatalytic activity of complexes **77** and **77a** for the conversion of CO_2_ into CO [124].

Photocatalyst ^a^	Light Source	CO, μmol (TON ^b^_CO_)	Rate, μmol⋅h^−1^ (TOF ^c^, h^−1^)
**77**	white LED	310 ± 30 (155 ± 16)	6.4 ± 0.6 (3.2 ± 0.3)
	(400 < λ < 750 nm)		
	(3.8 mW⋅cm^−2^)		
**77**	blue LED	344 ± 30 (172 ± 15)	4.6 ± 0.4 (2.3 ± 0.2)
	λ = 450 nm (fwhm 10 nm),		
	2 × 10^−8^ einstein⋅s^−1^		
	(1.07 mW⋅cm^−2^)		
**77**	blue LED	530 ± 50 (265 ± 25)	3.0 ± 0.4 (1.5 ± 0.2)
	λ = 450 nm (fwhm 10 nm),		
	7.9 × 10^−9^ einstein⋅s^−1^		
	(0.43 mW⋅cm^−2^)		
**77a**	white LED	178 ± 18 (89 ± 9)	16 ± 2 (8 ± 1)
	(400 < λ < 750 nm)		
	(3.8 mW⋅cm^−2^)		
**77a**	blue LED	182 ± 18 (91 ± 9)	9 ± 1 (4.5 ± 0.5)
	λ = 450 nm (fwhm 10 nm),		
	2 × 10^−8^ einstein⋅s^−1^		
	(1.07 mW⋅cm^−2^)		

**^a^** Reactions performed in triethanolamine/CH_3_CN (1/5 *v*/*v*) mixture (4 mL), under CO_2_-saturated conditions and in the presence of the photocatalyst (2 μmol). **^b^** Turnover number (TON_CO_) calculated from the maximum CO yield, in concomitance at the kinetics plateau. **^c^** Turnover frequency (TOF), calculated from the maximum rate of CO production, determined from a linear fit of the kinetic traces in the region of maximum slope.

## Data Availability

The datasets generated and/or analysed during the current study are available within the manuscript and Supplementary Materials.

## Supplementary Materials

The following supporting information can be downloaded at: https://www.mdpi.com/article/10.3390/molecules31111921/s1, Table S1. The absorption and emission properties of neutral Ir(III) complexes including pyrene and anthracene motifs alongside with the HOMO–LUMO energy gaps; Table S2. The absorption and emission properties of unsubstituted model chromophores of neutral Ir(III) complexes including pyrene and anthracene motifs; Table S3. The absorption and emission properties of cationic bis-cyclometalated iridium(III) complexes bearing pyrene-functionalized ligands alongside with the HOMO–LUMO energy gaps; Table S4. The absorption and emission properties of unsubstituted model chromophores of cationic bis-cyclometalated iridium(III) complexes bearing pyrene-functionalized ligands alongside with the HOMO–LUMO energy gaps; Table S5. The absorption and emission properties of cationic mono- and bis-cyclometalated iridium(III) complexes bearing anthracene-functionalized ligands alongside with the HOMO–LUMO energy gaps; Table S6. The absorption and emission properties of unsubstituted model chromophores of cationic mono- and bis-cyclometalated iridium(III) complexes bearing anthracene-functionalized ligands alongside with the HOMO–LUMO energy gaps. References [135,136,137,138,139,140,141,142] are cited in the Supplementary Materials.
