# Supplementary material for: Iridium(III) Complexes Bearing Pyrene- and Anthracene-Functionalized Ligands—Photophysics and Application Potential in Photocatalysis, Triplet-Triplet Annihilation Upconversion, Photodynamic Therapy, and Photoactivated Chemotherapy
Source: Molecules. 2026 Jun 2;31(11):1921. doi: 10.3390/molecules31111921 (PMC13258693; doi:10.3390/molecules31111921)
Supplement: Supplementary file 1 [file molecules-31-01921-s001.zip › molecules-4347817-supplementary.pdf]

SUPPORTING INFORMATION

Iridium(III) complexes bearing a pyrene and anthracene functionalized ligands – photophysics and application potential in photocatalysis, triplet-triplet annihilation up-conversion, photodynamic therapy and photoactivated chemotherapy

Anna Kryczka, Katarzyna Choroba*, Joanna Palion-Gazda, Barbara Machura*

Institute of Chemistry, University of Silesia, 9 Szkolna, 40-006 Katowice, Poland; anna.kryczka@us.edu.pl (A.K.); joanna.palion-gazda@us.edu.pl (J.P.-G.)

**Correspondence: katarzyna.choroba@us.edu.pl (K.C.); barbara.machura@us.edu.pl (B.M.)*

Table of Contents

Table S1. The absorption and emission properties of neutral Ir(III) complexes including pyrene and anthracene motifs alongside with the HOMO–LUMO energy gaps. 3

Table S2. The absorption and emission properties of unsubstituted model chromophores of neutral Ir(III) complexes including pyrene and anthracene motifs. 8

Table S3. The absorption and emission properties of cationic bis-cyclometalated iridium(III) complexes bearing pyrene-functionalized ligands alongside with the HOMO–LUMO energy gaps. 9

Table S4. The absorption and emission properties of unsubstituted model chromophores of cationic bis-cyclometalated iridium(III) complexes bearing pyrene-functionalized ligands alongside with the HOMO–LUMO energy gaps. 15

Table S5. The absorption and emission properties of cationic mono- and bis-cyclometalated iridium (III) complexes bearing anthracene-functionalized ligands alongside with the HOMO–LUMO energy gaps. 16

Table S6. The absorption and emission properties of unsubstituted model chromophores of cationic mono- and bis-cyclometalated iridium (III) complexes bearing anthracene-functionalized ligands alongside with the HOMO–LUMO energy gaps. 19

References 20

Table S1. The absorption and emission properties of neutral Ir(III) complexes including pyrene and anthracene motifs alongside with the HOMO–LUMO energy gaps.

Compound	medium	λ_{abs} [nm]	λ_{exc} [nm]	λ_{PL} [nm]	τ [µs]	φ_{PL}	E_g [eV]	Ref.
**1**	Toluene	~480, 465, 420, 380, 340, 330	390	510	τ_1= 0.0020<br>τ_2= 680	0.03	–	[1]
	MeTHF 77K	–	400	598	–	–	–	
**2**	Toluene	~480, 465, 420, 380, 340, 330	390	511	τ_1= 0.0024<br>τ_2= 2800	0.02	–	
	MeTHF 77K	–	400	589	–	–	–	
**3**	Toluene	~460, 410, 360, 340, 310	369<br>380	565	τ_1= 0.001<br>τ_2= 2200	0.66	–	[2]
	MeTHF 77K	–	380	589	–	–	–	
**4**	Toluene	~470, 410, 340, 330, 310	380	563	τ_1= 0.0105<br>τ_2= 1000	0.60	–	[3]
	MeTHF 77K	–	380	544	–	–	–	
	DCM	~450, 400, 350, 330, 320, 310, 290, 280	–	–	–	–	–	[4]
	Toluene	–	380	562	τ_1= 0.012<br>τ_2= 1000	0.59	–	
	MeTHF 77K	–	380	589	–	–	–	
**5**	Toluene	~460, 420, 340, 330, 310	380	552	τ_1= 0.0079<br>τ_2= 2000	0.31	–	[3]
	MeTHF 77K	–	380	527	–	–	–	
	DCM	~450, 400, 350, 330, 320, 310, 290, 280	–	–	–	–	–	[4]
	Toluene	–	380	553	τ_1= 0.0087<br>τ_2= 2000	0.19	–	
	MeTHF 77K	–	340	525	–	–	–	
**6**	Toluene	~460, 420, 360, 340, 330, 310	380	520	τ_1= 0.0074<br>τ_2= 3900	0.07	–	[3]
	MeTHF 77K	–	380	508	–	–	–	

**Compound**	**medium**	**λ_{abs} [nm]**	**λ_{exc} [nm]**	**λ_{PL} [nm]**	**τ [μs]**	**φ_{PL}**	**E_g [eV]**	**Ref.**
**7**	Toluene	~460, 420, 340, 330, 310	380	564	τ_1= 0.0040<br>τ_2= 1200	0.64	–	
	MeTHF 77K	–	380	550	–	–	–	
**8**	DMSO	347, 332, 280	366	388,<br>409,<br>505	1.0	<0 01	–	[5]
**9**	THF	390, 365, 295, 285, 248	460	420,<br>~520	–	–	–	[6]
	MeCN	–	–	–	0.0021	–		
**10**	DCM	408, 337, 277	410	563	–	<0.01	–	[7]
**11**		~450, 375, 350, 330, 290, 240	–	–	–	–	–	[8]
	PMMA	~400, 350, 330, 275	–	615	18	0.15	–	
**12**	Chlorobenzene	~475, 385, 375, 360	485	~570	–	–	3.20^{calc}<br>2.49^{exp}	[9]
**13**		~475, 380, 365, 350		~570	–	–	3.25^{calc}<br>2.44^{exp}	
**14**		~475, 410, 380, 360		~570	–	–	3.15^{calc}<br>2.46^{exp}	
**15**	DCM	454, 360, 259	–	471,<br>440	0.00188<br>0.00698	0.011	3.31^{calc}	[10]
**16**	2-MeTHF	620, 492, 410, 344, 283, 243	–	673	1.13	0.136	2.77^{calc}<br>2.25^{exp}	[11]
**17**		630, 494, 406, 350, 280, 243	–	665	1.18	0.189	2.81^{calc}<br>2.24^{exp}	
**18**	CH_3Cl	348, 276	–	425	0.0027	–	–	[12]
**19**		428, 348, 276	–	429	0.0028	–	–	
**20**		406, 348, 284	–	425	0.0025	–	2.17^{calc}	
**21**		415, 347, 278	–	446	0.0030	–	–	
**22**	DCM	519, 425sh, 404, 386sh, 309, 272	400	680,<br>734	2.7	0.0056	2.24^{calc}	[13]

Compound	medium	λ_{abs} [nm]	λ_{exc} [nm]	λ_{PL} [nm]	τ [μs]	φ_{PL}	E_g [eV]	Ref.
	Toluene	–		684, 734	2.5	0.0013		
	MeCN	–		680, 734	2.5	0.0088		
	EPA	–		680, 738	3.6	–		
	EPA 77K	–		679, 745	5.1	–		
**23**	DCM	493, 464, 432, 411, 321, 296, 274	430	626, 683	37.0	0.063	2.13^{calc}	
	Toluene	–		626, 680	52.9	0.021		
	MeCN	–		623, 685	11.6	0.060		
	EPA	–		623, 685	67.1	–		
	EPA 77K	–		622, 636, 676, 692	125.0	–		
**24**	DCM	~560, 440, 310	–	~705	–	–	–	[14]
**25**		545, 448, 426, 311	–	–	–	–	2.07^{exp}	[15]
**26**		524, 496, 403, 382sh, 311, 286	436	681	0.42	0.0031	3.02^{calc} 2.18^{exp}	[16]
	Hexane	406	–	680	–	–		
	Toluene	406	–	683	–	–		
	MeCN	399	–	676	–	–		
**27**	DCM	547, 513, 420, 304, 286	436	692	0.80	0.0033	2.96^{calc} 2.07^{exp}	
	Hexane	422	–	692	–	–		
	Toluene	424	–	695	–	–		
	MeCN	415	–	689	–	–		

Compound	medium	λ_{abs} [nm]	λ_{exc} [nm]	λ_{PL} [nm]	τ [μs]	φ_{PL}	E_g [eV]	Ref.
**28**	DCM	513, 480, 423sh, 399, 303,285	436	680	0.32	0.0026	3.08^{calc} 2.21^{exp}	
	Hexane	399	–	682	–	–		
	Toluene	401	–	682	–	–		
	MeCN	397	–	676	–	–		
**29**	DCM	509, 403, 384sh, 286	436	679	0.47	0.0024	3.08^{calc} 2.21^{exp}	
	Hexane	403	–	679	–	–		
	Toluene	406	–	682	–	–		
	MeCN	401	–	677	–	–		
**30**	DCM	511, 441, 416, 307, 240		698, 792sh	1.04	0.0225	2.25^{exp}	
	DCM 77K	–		693, 760sh	4.82	–		
**31**	DCM	511, 441, 417, 307, 237	440	699, 792sh	1.41	0.007	2.25^{exp}	[17]
	DCM 77K	–		702, 773sh	5.05	–		
**32**	DCM	511, 441, 417, 307, 237		700, 792sh	1.42	0.0122	2.25^{exp}	
	DCM 77K	–		704, 778sh	4.67	–		
**33**	DCM	608, 462, 415, 308	450	732	0.60	0.13	1.82^{exp}	[18]
	2-MeTHF 77K	–		706, 776	–	–		
	Toluene	608, 462, 415, 308		~715	–	–		
	THF	608, 462, 415, 308		~715	–	–		
	$CHCl_3$	608, 462, 415, 308		~750	–	–		
**34**	DCM	654, 472, 419	600	789	0.22	0.14	2.43^{calc} 1.85^{exp}	[19]
	2-MeTHF 77K	–		770, 859sh	–	–		

Compound	medium	λ_{abs} [nm]	λ_{exc} [nm]	λ_{PL} [nm]	τ [μs]	φ_{PL}	E_g [eV]	Ref.
	$CHCl_3$	~654, 472, 419		~790	–	–		
	THF	~654, 472, 419		~790	–	–		
	Toluene	~654, 472, 419		~790	–	–		

^{calc} – the HOMO–LUMO energy gaps determined by DFT calculations; ^{exp} – the HOMO–LUMO energy gaps determined by electrochemical measurements.

Table S2. The absorption and emission properties of unsubstituted model chromophores of neutral Ir(III) complexes including pyrene and anthracene motifs.

Compound	medium	λ_{abs} [nm]	λ_{exc} [nm]	λ_{PL} [nm]	τ [µs]	φ_{PL}	Ref.
**1a = 2a**	Toluene	~480, 465, 420, 380, 340, 330	390	510	1.3	0.97	[1]
	MeTHF 77K	–	400	496	–	–	
**3a**	Toluene	~460, 410, 360, 340, 310	369	565	2.2	0.92	[2]
	MeTHF 77K	–	380	546	–	–	
**4a**	Toluene	~470, 410, ~335	369	560	2.2	0.92	[3]
	MeTHF 77K	–	380	545	–	–	
	DCM	~450, 400, 340	–	–	–	–	[4]
	Toluene	–	380	561	2.3	0.92	
**5a**	Toluene	~450, 400, 340	380	550	3.8	0.78	[3]
	MeTHF 77K	–	380	530	–	–	
	DCM	~450, 400, 340	–	–	–	–	[4]
	Toluene	–	380	550	3.8	0.81	
**6a**	Toluene	~460, 420, 340	369	517	1.4	0.97	[3]
	MeTHF 77K	–	380	512	–	–	
**7a**	Toluene	~460, 420, 340, 320, 310	369	563	2.9	0.90	
	MeTHF 77K	–	380	547	–	–	

Table S3. The absorption and emission properties of cationic bis-cyclometalated iridium(III) complexes bearing pyrene-functionalized ligands alongside with the HOMO–LUMO energy gaps.

Compound	medium	λ_{abs} [nm]	k_r (s^{-1})	k_{nr} (s^{-1})	λ_{exc} [nm]	λ_{PL} [nm]	τ [μs]	τ_{TA}[μs]	φ_{PL}	E_g [eV]	Ref.
**35**	MeCN	377	–	–	420	648, ~710	–	53.3	–	–	[20]
**36**		410	–	–		671, ~740	–	60.1	–		
**37**		414	–	–		679, ~750	–	60.5	–		
**38**	$CHCl_3$	~425, 350, 330, 315, 280, 265	–	–	350	~400, 425, 560	0.0018 (97%)<br>0.520 (3%)	–	–	–	[21]
**39**	DCM	~425, 370, 350, 325, 315, 280, 275, 240	$7.75 \cdot 10^5$	$2.49 \cdot 10^8$	~450	668, 740 (sh)	0.004	–	0.003	2.89^{calc}	[22]
	DCM 77K	–	–	–	380	663, 725	~1	–	–		
**40**	DCM	~440, 370, 360, 350, 325, 315, 280, 275, 240	$8.63 \cdot 10^5$	$2.42 \cdot 10^8$	–	665, 740 (sh)	0.007	–	0.006	2.83^{calc}	
	DCM 77K	–	–	–	380	663, 725	~1	–	–		
**41**	MeCN	~450, 420, 350, 330, 315, 275, 260, 250, 240	$4.22 \cdot 10^2$	$4.02 \cdot 10^3$	465	590, 625	225	225	0.095	–	[23]
	77K	–	–	–	413	~600, 615, 650, 660	–	–	–		
	MeCN	~450, 420, 350, 330, 315, 275, 260, 250, 240	$4.22 \cdot 10^2$	$4.02 \cdot 10^3$	–	590, 625	225	–	0.095	–	[24]
	BuCN 77K	–	–	–	413	~600, 615, 650, 660	–	–	–		
**42**	MeCN	~450, 420, 350, 330, 315, 275, 260, 250, 240	$2.00 \cdot 10^2$	$1.88 \cdot 10^3$	–	590, 625	480	–	0.096	–	
	BuCN 77K	–	–	–	413	~600, 615, 650, 660	–	–	–		
**43**	MeCN	408	–	–	420	683, ~750	–	56.7	–	–	[20]
**44**		~475, 400, 325, 290	–	–	400	–	–	–	–	–	[25]
**45**		411	–	–	420	667, ~740	–	92.4	–	–	[20]
	DCM	440, 414	$7.35 \cdot 10^1$	$7.27 \cdot 10^3$	440	672, 747	136.1	157.2	0.01	–	[26]

Compound	medium	λ_{abs} [nm]	k_r (s^{-1})	k_{nr} (s^{-1})	λ_{exc} [nm]	λ_{PL} [nm]	τ [μs]	τ_{TA}[μs]	φ_{PL}	E_g [eV]	Ref.
	77K	–	–	–		~670, 740	–	–	–		
**46**	DCM	454, 481	$1.78\cdot10^{2}$	$1.35\cdot10^{4}$	480	682, 757	73.1	85.8	0.013	–	
	77K	–	–	–		~670, 760	–	–	–		
**47**	DCM	410, 434	4.69	$4.69\cdot10^{3}$	440	678, 751	213.1	367.7	0.001	–	
	77K	–	–	–		~660, 725	–	–	–		
**48**	DCM	445, 485	$3.85\cdot10^{3}$	$7.65\cdot10^{5}$		600, 738	1.3	247.1	0.005	–	
	77K	–	–	–		~580, 760	–	–	–		
**49**	DCM	482	$5.83\cdot10^{2}$	$2.10\cdot10^{4}$	482	677	46.3	53.3	0.027	–	[27]
	EtOH/MeOH (4:1, v/v) 77K	–	–	–		~670, 750	122.8	–	–		
**50**	DCM	485	$3.47\cdot10^{1}$	$5.75E\cdot10^{3}$	440	680, ~760 (sh)	172.8	195.5	0.006	–	[28]
	DCM 77K	–	–	–	–	~670, 750	586.4	–	–		
**51**	DCM	486	$1.16\cdot10^{2}$	$1.28\cdot10^{4}$	440	685, ~770 (sh)	77.5	72.8	0.009	–	
	DCM 77K	–	–	–	–	~670, 750	472.7	–	–		
**52**	DCM	486	$1.04\cdot10^{2}$	$1.48\cdot10^{4}$	440	690, ~770 (sh)	67.2	68.5	0.007	–	
	DCM 77K	–	–	–	–	~685	261.6	–	–		
**53**	MeCN	368	$1.00\cdot10^{3}$	$8.69\cdot10^{5}$	375	574	–	5.08	0.0011	3.14^{calc}	[29]
**55**		448sh, 415, 398, 382, 283sh	–	–	–	548	0.60	–	–	3.42^{calc}	[30]
	2-MeTHF 77K	–	–	–	–	658, 721	–	–	–		
	Toluene	–	–	–	–	–	–	28.5	0.68		
	Toluene / 10% DCM	~430, 410, 380	–	–	436	541, 664	–	–	–		
	THF	~430, 410, 380	–	–		585, 665, 730	–	–	–		
	DCM	~430, 410, 380	–	–		577, 664	–	–	–		
	Acetone	~448sh, 415, 398, 382, 283sh	–	–		548, 659	–	–	–		
	MeCN	~430, 410, 380	–	–		548	0.6	–	–		
**56**	PMMA	–	–	–	–	631	0.329<br>2.4	0.378<br>2.9	0.004	–	[8]
	DCM	~470, 400, 350, 330, 310, 280, 250	–	–	–	–	–	–	–		
**57**		~470, 400, 350, 330, 310, 280, 250	$5.19\cdot10^{3}$	$3.65\cdot10^{5}$	–	615	2.7	3.0	0.014	–	

**Compound**	**medium**	**λ_{abs} [nm]**	**k_r (s^{-1})**	**k_{nr} (s^{-1})**	**λ_{exc} [nm]**	**λ_{PL} [nm]**	**τ [μs]**	**τ_{TA}[μs]**	**φ_{PL}**	**E_g [eV]**	**Ref.**
	PMMA	–	–	–	–	610	2.0<br>28.1	0.378<br>2.9	0.028		
**58**	DCM/toluene (1:3) 77K	–	–	–	280	626, 642, 679, 696, 716, 764, 824	–	–	–	–	[31]
	MeCN	~400, 360, 350, 300	–	–	–	–	–	22	–		
**59**	DCM/toluene (1:3) 77K	–	–	–	450	626, 643, 658, 680, 697, 716, 763, 824	–	–	–		
	MeCN	~450, 390, 380, 280	–	–	–	–	–	31	–		
**60**	DCM/toluene (1:3) 77K	–	–	–	330	615, 678, 750sh	–	–	–		
	MeCN	~350, 330, 280	–	–		427	0.01	22	<0.001		
**61**	DCM/toluene (1:3) 77K	–	–	–		616, 670	–	–	–		
	MeCN	~450, 350, 300	–	–		439	0.013	18	<0.001		
**62**	DCM/toluene (1:3) 77K	–	–	–		613, 676, 736sh	–	–	–		
	MeCN	~420, 350, 320, 300	–	–		403	0.011	2.4	<0.001		
**63**	DCM	~525, 450, 380, 350, 300, 280	$4.5 \cdot 10^3$	$3.2 \cdot 10^5$	410	704, 771sh	–	–	–	–	[32]
	THF	–	$4.50 \cdot 10^2$	$3.21 \cdot 10^5$		–	3.11	–	0.0014		
**64**	$CHCl_3$	–	–	–	–	435	0.0027	13.3	–	–	[12]
**65**		–	–	–	–	407	0.0026	3.9	–	–	
**66**	DCM	507, 402, 384, 356, 338, 290	$1.95 \cdot 10^5$	$5.21 \cdot 10^6$	–	651	0.185	–	0.036	–	[33]
	MeCN	507, 402, 384, 356, 338, 290	$1.17 \cdot 10^5$	$8.22 \cdot 10^6$	–	655	0.12	0.119<br>0.110<br>0.108	0.014		
	THF	507, 402, 384, 356, 338, 290	$1.53 \cdot 10^5$	$5.73 \cdot 10^6$	–	649	0.17	–	0.026		
	Toluene (10% DCM)	507, 402, 384, 356, 338, 290	$1.58 \cdot 10^5$	$8.18 \cdot 10^6$	–	654	0.12	–	0.019		

Compound	medium	λ_{abs} [nm]	k_r (s^{-1})	k_{nr} (s^{-1})	λ_{exc} [nm]	λ_{PL} [nm]	τ [μs]	τ_{TA}[μs]	φ_{PL}	E_g [eV]	Ref.
**67**	DCM	544, 402, 381, 355, 340, 290	$6.57\cdot10^4$	$1.42\cdot10^7$	–	710	0.07	–	0.0046	–	
	MeCN	544, 402, 381, 355, 340, 290	$4.67\cdot10^4$	$3.33\cdot10^7$	–	714	0.03	0.055<br>0.056	0.0014		
	THF	544, 402, 381, 355, 340, 290	$8.25\cdot10^4$	$2.49\cdot10^7$	–	705	0.04	–	0.0033		
	Toluene (10% DCM)	544, 402, 381, 355, 340, 290	$5.80\cdot10^4$	$1.99\cdot10^7$	–	712	0.05	–	0.0029		
**68**	DCM	605, 404, 384, 356, 325, 290	–	–	–	810	0.38	–	–	–	
	MeCN	605, 404, 384, 356, 325, 290	–	–	–	803	0.32	0.392<br>0.405<br>0.380	–		
	THF	605, 404, 384, 356, 325, 290	–	–	–	808	0.24	–	–		
	Toluene (10% DCM)	605, 404, 384, 356, 325, 290	–	–	–	791	0.28	–	–		
**69**	DCM	498, 406, 340, 290	$1.38\cdot10^5$	$9.26\cdot10^5$	–	625	0.94	–	0.13	3.07^{calc}<br>2.40^{exp}	[34]
	MeCN	498, 406, 340, 290	$8.82\cdot10^4$	$1.38\cdot10^6$	–	628	0.68	0.65<br>0.65<br>0.67	0.06		
	THF	498, 406, 340, 290	$1.55\cdot10^5$	$1.04\cdot10^6$	–	624	0.84	–	0.13		
	Toluene (10% DCM)	498, 406, 340, 290	$1.70\cdot10^5$	$1.96\cdot10^6$	–	622	0.47	–	0.08		
**70**	DCM	445, 401, 339, 292	$1.13\cdot10^5$	$6.55\cdot10^6$	–	593	0.15	–	0.017	3.20^{calc}<br>2.48^{exp}	
	MeCN	445, 401, 339, 292	–	–	–	600	–	15.3<br>15.0<br>16.1	0.0089		
	THF	445, 401, 339, 292	–	–	–	600	–	–	0.0097		
	Toluene (10% DCM)	445, 401, 339, 292	–	–	–	598	–	–	0.0096		
**71**	DCM	580, 496, 405, 344, 290	$1.29\cdot10^5$	$9.46\cdot10^5$	–	657	0.93	–	0.12	2.86^{calc}<br>2.24^{exp}	

Compound	medium	λ_{abs} [nm]	k_r (s^{-1})	k_{nr} (s^{-1})	λ_{exc} [nm]	λ_{PL} [nm]	τ [μs]	τ_{TA}[μs]	φ_{PL}	E_g [eV]	Ref.
	MeCN	580, 496, 405, 344, 290	$1.09\cdot10^5$	$2.11\cdot10^6$	–	663	0.45	0.56<br>0.46<br>0.47	0.049		
	THF	580, 496, 405, 344, 290	$9.83\cdot10^4$	$1.63\cdot10^6$	–	657	0.58	–	0.057		
	Toluene (10% DCM)	580, 496, 405, 344, 290	$1.00\cdot10^5$	$4.90\cdot10^6$	–	672	0.20	–	0.020		
**72**	DCM	485, 384, 328, 291	$6.34\cdot10^4$	$6.83\cdot10^5$	–	645	1.34	–	0.085	2.97^{calc}<br>2.45^{exp}	
	MeCN	485, 384, 328, 291	–	–	–	–	–	13.1<br>14.9<br>13.9	–		
	THF	485, 384, 328, 291	$3.18\cdot10^4$	$7.43\cdot10^5$	–	645	1.29	–	0.041		
	Toluene (10% DCM)	485, 384, 328, 291	$8.33\cdot10^4$	$2.69\cdot10^6$	–	645	0.36	–	0.030		
**73**	DCM	550, 469, 444, 405, 357, 309, 292	$2.22\cdot10^3$	$4.50\cdot10^5$	–	740	2.21	–	0.0049	2.82^{calc}<br>2.20^{exp}	
	MeCN	550, 469, 444, 405, 357, 309, 292	$1.33\cdot10^3$	$4.73\cdot10^5$	–	735	2.11	2.80<br>2.68<br>2.82	0.0028		
	THF	550, 469, 444, 405, 357, 309, 292	$2.00\cdot10^3$	$5.11\cdot10^5$	–	739	1.95	–	0.0039		
	Toluene (10% DCM)	550, 469, 444, 405, 357, 309, 292	$2.24\cdot10^3$	$4.95\cdot10^5$	–	731	2.01	–	0.0045		
**74**	DCM	599, 480, 402, 386, 336, 292	$4.53\cdot10^3$	$1.16\cdot10^6$	–	766	0.86	–	0.0039	2.70^{calc}<br>2.14^{exp}	
	MeCN	599, 480, 402, 386, 336, 292	$3.38\cdot10^3$	$1.41\cdot10^6$	–	766	0.71	0.86<br>0.83<br>0.87	0.0024		
	THF	599, 480, 402, 386, 336, 292	$3.68\cdot10^3$	$1.15\cdot10^6$	–	765	0.87	–	0.0032		
	Toluene (10% DCM)	599, 480, 402, 386, 336, 292	$6.76\cdot10^3$	$2.70\cdot10^6$	–	765	0.37	–	0.0025		
**75**	Toluene	403	–	–	404	569		56.1	0.076	2.06^{calc}	[35]

**Compound**	**medium**	**λ_{abs} [nm]**	**k_r (s^{-1})**	**k_{nr} (s^{-1})**	**λ_{exc} [nm]**	**λ_{PL} [nm]**	**τ [µs]**	**τ_{TA}[µs]**	**φ_{PL}**	**E_g [eV]**	**Ref.**
	EtOH/MeOH (4:1 v/v) 77K	–	–	–		~560, 575, 625, 680	–	–	–		
**76**	Toluene	466	–	–	460	607		73.9	0.034	2.30^{calc}	
	EtOH/MeOH (4:1 v/v) 77K	–	–	–		~600, 650, 720	–	–	–		

^{calc} – the HOMO–LUMO energy gaps determined by DFT calculations; ^{exp} – the HOMO–LUMO energy gaps determined by electrochemical measurements.

Table S4. The absorption and emission properties of unsubstituted model chromophores of cationic bis-cyclometalated iridium(III) complexes bearing pyrene-functionalized ligands alongside with the HOMO–LUMO energy gaps.

**Compound**	**medium**	**λ_{abs} [nm]**	**k_r (s^{-1})**	**k_{nr} (s^{-1})**	**λ_{exc} [nm]**	**λ_{PL} [nm]**	**τ [µs]**	**τ_{TA}[µs]**	**φ_{PL}**	**E_g [eV]**	**Ref.**
**35a = 36a = 37a = 39a**	MeCN	300	–	–	420	585	–	0.3	–	–	[20]
	DCM	~425, 365, 340, 320, 310, 275, 260, 240	$3.37\cdot10^{5}$	$1.45\cdot10^{6}$	380	595	0.561	–	0.189		[22]
	77K	–	–	–		520, 552	3.5	–	–		
**38a**	$CHCl_3$	~425, 345, 310, 260	–	–	350	~560	0.78	–	–	–	[21]
**40a**	DCM	~425, 365, 340, 320, 310, 275, 260, 240	$4.32\cdot10^{5}$	$3.5\cdot10^{5}$	380	535	1.279	–	0.552	–	[22]
	DCM 77K	–	–	–		475, 504	–	–	–		
**41a = 42a**	MeCN	~450, 420, 330, 310, 275, 210	$1.20\cdot10^{3}$	$1.19\cdot10^{5}$	465	590, 625	8.3	–	0.01	–	[23] [24]
**45a = 46a**	DCM	523, 479, 408, 379, 265, 226	$7.20\cdot10^{5}$	$1.34\cdot10^{6}$	–	566	0.4859	–	0.35	2.64^{exp}	[36]
	Solid	–	–	–	–	584	–	–	0.116		
**47a**	DCM	410, 377, 333, 268, 255	$1.20\cdot10^{5}$	$1.96\cdot10^{5}$	260	559	–	3.17	0.38	3.15^{calc}	[37]
**48a**		377, 269, 250, 242	$1.73\cdot10^{5}$	$6.14\cdot10^{5}$		568	–	1.27	0.22	2.74^{exp}	
**50a = 51a = 52a**	DCM/MeOH (9:1 v/v)	485	$2.55\cdot10^{4}$	$1.16\cdot10^{5}$	–	589	7.06	–	0.18	2.23^{exp}	[38]
	DCM/MeOH (9:1 v/v) 77K	–	–	–	–	579	87.1	–	–		
**53a = 54a**	DCM	416, 390, 348, 299, 279	$3.41\cdot10^{5}$	$1.08\cdot10^{6}$	315	568	0.7043	–	0.24	2.87^{calc}	[39]
**55a = 94a**	MeCN	432, 392, 377, 336, 270	$5.7\cdot10^{5}$	$2.1\cdot10^{6}$	436	600	0.37	–	0.21	3.54^{calc}	[30]
	2-MeTHF 77K	–	–	–		524	6.37	–	–		
	solid	–	–	–		584	–	–	–		
	Toluene	–	–	–		–	–	0.36	–		
	Toluene/10% DCM	~460, 405, 390	$7.50\cdot10^{5}$	$1.75\cdot10^{6}$		600	0.4	–	0.30		
	THF	~460, 400, 380	$5.85E\cdot10^{5}$	$1.85\cdot10^{6}$		600	0.41	–	0.24		
	DCM	~500, 460, 410, 390	$8.86E\cdot10^{5}$	$5.43\cdot10^{5}$		584	0.7	–	0.62		
	Acetone	~460, 400, 380	$5.83\cdot10^{5}$	$2.19E\cdot10^{6}$		600	0.36	–	0.21		
**56a = 57a**	PMMA	–	–	–		583	0.046	–	0.032	–	[8]

Compound	medium	λ_{abs} [nm]	k_r (s^{-1})	k_{nr} (s^{-1})	λ_{exc} [nm]	λ_{PL} [nm]	τ [μs]	τ_{TA}[μs]	φ_{PL}	E_g [eV]	Ref.
							0.198				
	DCM	~480, 375, 340	–	–	–	–	–	–	–		
**60a**	MeCN	~390, 350, 290	$1.77·10^5$	$2.77·10^5$	310	536, 568	2.2	9.0	0.39	–	[40]
	Toluene:DCM (1:3) 77K	–	–	–		~525, 570, 610	–	–	–		
**62a**	MeCN	~420, 400	$1.29·10^5$	$4.59·10^5$		542, 572	1.7	1.8	0.22		
	Toluene:DCM (1:3) 77K	–	–	–		~540, 580, 620	–	–	–		
^{calc} – the HOMO–LUMO energy gaps determined by DFT calculations; ^{exp} – the HOMO–LUMO energy gaps determined by electrochemical measurements.											

Table S5. The absorption and emission properties of cationic mono- and bis-cyclometalated iridium (III) complexes bearing anthracene-functionalized ligands alongside with the HOMO–LUMO energy gaps.

Compound	medium	λ_{abs} [nm]	k_r (10^5 s^{-1})	k_{nr} (10^5 s^{-1})	λ_{exc} [nm]	λ_{PL} [nm]	τ [μs]	τ_{TA}[μs]	φ_{PL}	E_g [eV]	Ref.
**77**	MeCN	505sh, 383, 364, 328, 313, 284sh, 273, 253	0.25	2.69	380	553	3.41	–	0.0844	2.23^{calc}	[41]
**78**	DMSO:PBS (0.5:99.5 v/v)	477, 454, 387, 368	–	–	460	512	–	–	–	–	[42]
**79**	MeCN	384, 359, 331, 317, 290, 253	–	–	330	545	0.0361 (3%) 0.23 (97%)	–	0.018	2.50^{calc} 2.45^{exp}	[43]
	H_2O:DMSO (99:1)	386, 337, 320 290, 254	–	–	–	–	–	–	–		
**80**	MeOH	375	–	–	730*	565	–	–	0.017	–	[44]
**81**	MeCN	387	17400	26000	–	440	0.23	–	0.40	2.70^{calc}	[45]
**82**	1% DMSO/H_2O	~380, 320, 280, 250	–	–	450	~580	0.037	–	–	–	[46]
**83**	Carbonate buffer (0.1 M, pH = 10.5)	386, 365, 647	–	–	–	625	–	–	<0.01	–	[47]
**83^{ep}**		–	–	–	–	625, I/I_o=20	–	–	–		
**84**		386, 365, 647	–	–	–	640	–	–	<0.01		

Compound	medium	λ_{abs} [nm]	k_r (10^5 s^{-1})	k_{nr} (10^5 s^{-1})	λ_{exc} [nm]	λ_{PL} [nm]	τ [µs]	τ_{TA}[µs]	φ_{PL}	E_g [eV]	Ref.
**84**^{ep}	containing Na_2MoO_4 (without addition of H_2O_2 for 83, 84 and after addition of H_2O_2 for 83^{ep}, 84^{ep}	–	–	–	–	640, I/I_o=15	–	–	–		
**85**	DMSO	424, 395, 375, 354, 320, 260	–	–	437	486, 545, 551	0.186	–	0.083	–	[48]
**86**		423, 394, 374, 353, 324, 260	–	–	432	521, 551	0.242	–	0.064	–	
**87**	MeCN	~390, 290, 253	6.53	13.88	382	438, 572	0.049	–	0.32	–	[49]
**88**	DCM	388, 369, 288, 256	–	–	–	–	–	–	–	–	[50]
**88**^{ep}		–	–	–	–	578	0.122, 0.223	–	0.125	–	
		–	–	–	–	+TFA: 582	0.067, 0.210	–	–		
**89**		388, 369, 284, 256	–	–	–					–	
**89**^{ep}		–	–	–	–	519	0.138, 0.468	–	0.372	–	
		–	–	–	–	+TFA: 528	0.271, 0.495	–	–		
**90**	DCM	470, 387, 368, 350, 324, 311, 291, 281, 257	–	–	397	–	–	–	–	–	[51]
**90**^{ep}						~525, 565, 615				–	
**91**		470, 389, 370, 351, 324, 311, 295, 256	–	–		–	–	–	–	–	
**91**^{ep}		–	–	–		~525, 560, 615	–	–	–	–	
**92**	MeOH	403	–	–	405	592	0.365*	–	–	–	[52]
**92**^{ep}		–	–	–		592	0.874**	–	–	–	
**93**		392	–	–		608	0.452**	–	–	–	
**93**^{ep}		–	–	–		608	0.630**	–	–	–	
**94**	MeCN	464, 387, 368, 253	0.18	25	436	590	0.390	–	0.007	3.43^{calc}	[30]

**Compound**	**medium**	**λ_{abs} [nm]**	**k_r (10^5 s^{-1})**	**k_{nr} (10^5 s^{-1})**	**λ_{exc} [nm]**	**λ_{PL} [nm]**	**τ [μs]**	**τ_{TA}[μs]**	**φ_{PL}**	**E_g [eV]**	**Ref.**
	Toluene/ 10% DCM	~464, 387, 368, 253	–	–		588	0.390	–	0.004		
	THF	~464, 387, 368, 253	–	–		589	0.600	–	0.13		
	DCM	~464, 387, 368, 253	–	–		580	0.690	–	0.015		
	Acetone	~464, 387, 368, 253	–	–		590	0.390	–	0.008		
	2-MeTHF 77K	–	–	–		555<br>600	4.62<br>3.89	–	–		
	Toluene	–	–	–	355	–	–	24.4<br>3.57,<br>25.0	–		

* At the maximum of two-photon absorption cross-section; ** The lifetime was recorded under hypoxia; ep endoperoxide form; I/I_o – The emission-enhancement factors; calc – the HOMO–LUMO energy gaps determined by DFT calculations; exp – the HOMO–LUMO energy gaps determined by electrochemical measurements.

Table S6. The absorption and emission properties of unsubstituted model chromophores of cationic mono- and bis-cyclometalated iridium (III) complexes bearing anthracene-functionalized ligands alongside with the HOMO–LUMO energy gaps.

Compound	medium	λ_{abs} [nm]	k_r (s^{-1})	k_{nr} (s^{-1})	λ_{exc} [nm]	λ_{PL} [nm]	τ [μs]	τ_{TA}[μs]	φ_{PL}	E_g [eV]	Ref.
**77a**	MeCN	499, 389, 328, 301, 279, 270, 234	$1.15 \cdot 10^5$	$1.42 \cdot 10^5$	380	541	3.88	–	0.45	–	[41]
**78a**	DMSO:PBS (0.5:99.5 v/v)	479, 452, 369	–	–	460	514	–	–	–	–	[42]
**80a = 81a**	MeOH	365	–	–	–	550	–	–	0.185	–	[43]
**88a**	MeCN	462, 387, 333, 314, 288, 252	$2.86 \cdot 10^5$	$1.06 \cdot 10^6$	387, 371, 292, 250	601	0.743	0.612	0.2123	3.61^{calc} 2.51^{exp}	[53]
	DCM	504, 473, 390, 288, 268, 252	$2.81 \cdot 10^5$	$4.91 \cdot 10^5$	392, 372, 292, 254	591	1.296	–	0.3642		
	EtOH:MeOH (4:1 v/v) 77K	–	–	–	433, 407, 388, 368, 291	534	6.539	–	–		
	Solid	–	–	–	466, 364	560	0.971	–	0.0805		
**90a**	MeCN	503, 427, 368, 344, 316, 283, 268, 253, 234	$8.76 \cdot 10^4$	$1.73 \cdot 10^5$	389, 310, 292, 251	525, 563, 605	3.84	3.148	0.3362	3.70^{calc} 3.46^{exp}	
	DCM	519, 438, 397, 372, 316, 288, 254	$1.06 \cdot 10^5$	$1.83 \cdot 10^5$	430, 396, 312, 293, 254	522, 562, 602	3.467	–	0.3664		
	EtOH:MeOH (4:1 v/v) 77K	–	–	–	434, 409, 392, 372, 317, 295	514, 526, 554, 570, 603, 658	11.663	–	–		
	Solid	–	–	–	512, 488, 368, 311	576, 619	0.752	–	0.0336		
**92a = 93a**	DMSO/PBS (v/v = 1:999)	253, 286, 383	$1.14 \cdot 10^6$	$9.44 \cdot 10^6$	405	598	0.0945	–	0.108	–	[54]

^{calc} – the HOMO–LUMO energy gaps determined by DFT calculations; ^{exp} – the HOMO–LUMO energy gaps determined by electrochemical measurements.

References

1. Jiang, X.; Guo, X.; Peng, J.; Zhao, D.; Ma, Y. Triplet–Triplet Annihilation Photon Upconversion in Polymer Thin Film: Sensitizer Design. *ACS Appl. Mater. Interfaces* **2016**, *8*, 11441–11449, doi:10.1021/acsami.6b01339.
2. Guo, X.; Chen, Q.; Tong, Y.; Li, Y.; Liu, Y.; Zhao, D.; Ma, Y. Enhanced Triplet Sensitizing Ability of an Iridium Complex by Intramolecular Energy-Transfer Mechanism. *J. Phys. Chem. A* **2018**, *122*, 6963–6969, doi:10.1021/acs.jpca.8b04807.
3. Jiang, X.; Peng, J.; Wang, J.; Guo, X.; Zhao, D.; Ma, Y. Iridium-Based High-Sensitivity Oxygen Sensors and Photosensitizers with Ultralong Triplet Lifetimes. *ACS Appl. Mater. Interfaces* **2016**, *8*, 3591–3600, doi:10.1021/acsami.5b07860.
4. Peng, J.; Jiang, X.; Guo, X.; Zhao, D.; Ma, Y. Sensitizer Design for Efficient Triplet–Triplet Annihilation Upconversion: Annihilator-Appended Tris-Cyclometalated Ir(III) Complexes. *Chem. Commun.* **2014**, *50*, 7828–7830, doi:10.1039/C4CC01465K.
5. Kazama, A.; Imai, Y.; Okayasu, Y.; Yamada, Y.; Yuasa, J.; Aoki, S. Design and Synthesis of Cyclometalated Iridium(III) Complexes—Chromophore Hybrids That Exhibit Long-Emission Lifetimes Based on a Reversible Electronic Energy Transfer Mechanism. *Inorg. Chem.* **2020**, *59*, 6905–6922, doi:10.1021/acs.inorgchem.0c00363.
6. Spaenig, F.; Olivier, J.-H.; Prusakova, V.; Retailleau, P.; Ziessel, R.; Castellano, F.N. Excited-State Properties of Heteroleptic Iridium(III) Complexes Bearing Aromatic Hydrocarbons with Extended Cores. *Inorg. Chem.* **2011**, *50*, 10859–10871, doi:10.1021/ic201397v.
7. Dang, T.T.; Bonneau, M.; Gareth Williams, J.A.; Le Bozec, H.; Doucet, H.; Guerchais, V. Pd-Catalyzed Functionalization of the Thenoyltrifluoroacetone Coligands by Aromatic Dyes in Bis(Cyclometallated) IrIII Complexes: From Phosphorescence to Fluorescence?-. *European Journal of Inorganic Chemistry* **2015**, *2015*, 2956–2964, doi:10.1002/ejic.201500227.
8. Howarth, A.J.; Davies, D.L.; Lelj, F.; Wolf, M.O.; Patrick, B.O. Tuning the Emission Lifetime in Bis-Cyclometalated Iridium(III) Complexes Bearing Iminopyrene Ligands. *Inorg. Chem.* **2014**, *53*, 11882–11889, doi:10.1021/ic501032t.
9. Witkowska, E.; Orwat, B.; Oh, M.J.; Wiosna-Salyga, G.; Glowacki, I.; Kownacki, I.; Jankowska, K.; Kubicki, M.; Gierczyk, B.; Dutkiewicz, M.; et al. Effect of β-Ketoiminato Ancillary Ligand Modification on Emissive Properties of New Iridium Complexes. *Inorg. Chem.* **2019**, *58*, 15671–15686, doi:10.1021/acs.inorgchem.9b02785.
10. Maity, A.; Sarkar, R.; Rajak, K.K. Heteroleptic Iridium(III) Complexes Bearing a Coumarin Moiety: An Experimental and Theoretical Investigation. *RSC Adv.* **2015**, *5*, 78852–78863, doi:10.1039/C5RA08349D.
11. Hao, Z.; Zhang, K.; Wang, P.; Lu, X.; Lu, Z.; Zhu, W.; Liu, Y. Deep Red Iridium(III) Complexes Based on Pyrene-Substituted Quinoxaline Ligands for Solution-Processed Phosphorescent Organic Light-Emitting Diodes. *Inorg. Chem.* **2020**, *59*, 332–342, doi:10.1021/acs.inorgchem.9b02477.
12. Hallett, A.J.; White, N.; Wu, W.; Cui, X.; Horton, P.N.; Coles, S.J.; Zhao, J.; Pope, S.J.A. Enhanced Photooxidation Sensitizers: The First Examples of Cyclometalated Pyrene Complexes of Iridium(III). *Chem. Commun.* **2012**, *48*, 10838–10840, doi:10.1039/C2CC35907C.
13. Edkins, R.M.; Fucke, K.; Peach, M.J.G.; Crawford, A.G.; Marder, T.B.; Beeby, A. Syntheses, Structures, and Comparison of the Photophysical Properties of Cyclometalated Iridium Complexes Containing the Isomeric 1- and 2-(2′-Pyridyl)Pyrene Ligands. *Inorg. Chem.* **2013**, *52*, 9842–9860, doi:10.1021/ic400819f.
14. Williams, E.L.; Li, J.; Jabbour, G.E. Organic Light-Emitting Diodes Having Exclusive near-Infrared Electrophosphorescence. *Appl. Phys. Lett.* **2006**, *89*, 083506, doi:10.1063/1.2335275.
15. Lee, W.; Kwon, T.-H.; Kwon, J.; Kim, J.; Lee, C.; Hong, J.-I. Effect of Main Ligands on Organic Photovoltaic Performance of Ir(III) Complexes. *New J. Chem.* **2011**, *35*, 2557–2563, doi:10.1039/C1NJ20446G.

16. Liu, S.; Wang, M.; Wang, Y.; Hou, T.; Shen, X. Novel Deep Red to Near-Infrared Phosphorescent Iridium(III) Complexes Bearing Pyrenyl: Syntheses, Structures and Modulation of the Photophysical Properties. *Inorganic Chemistry Communications* **2023**, *150*, 110460, doi:10.1016/j.inoche.2023.110460.
17. Hao, Z.; Li, M.; Liu, Y.; Wang, Y.; Xie, G.; Liu, Y. Near-Infrared Emission of Dinuclear Iridium Complexes with Hole/Electron Transporting Bridging and Their Monomer in Solution-Processed Organic Light-Emitting Diodes. *Dyes and Pigments* **2018**, *149*, 315–322, doi:10.1016/j.dyepig.2017.09.061.
18. You, C.; Liu, D.; Meng, F.; Wang, Y.; Yu, J.; Wang, S.; Su, S.; Zhu, W. Iridium(III) Phosphors with Rigid Fused-Heterocyclic Chelating Architectures for Efficient Deep-Red/near-Infrared Emissions in Polymer Light-Emitting Diodes. *J. Mater. Chem. C* **2019**, *7*, 10961–10971, doi:10.1039/C9TC03645H.
19. Li, M.; Wang, L.; You, C.; Liu, D.; Zhang, K.; Zhu, W. Azaacene Containing Iridium(III) Phosphors: Elaboration of the π-Conjugation Effect and Application in Highly Efficient Solution-Processed near-Infrared OLEDs. *Dalton Trans.* **2023**, *52*, 16276–16284, doi:10.1039/D3DT02629A.
20. Wang, P.; Guo, S.; Zhao, Q.-P.; Xu, S.-Y.; Lv, H.; Lu, T.-B.; Zhang, Z.-M. Identification of Crucial Photosensitizing Factors to Promote CO2-to-CO Conversion. *Angewandte Chemie International Edition* **2024**, *63*, e202312450, doi:10.1002/anie.202312450.
21. Aboshi, R.; Takizawa, S.; Murata, S. Visible-Light-Driven Electron Transport across Vesicle Membrane Sensitized by Cationic Iridium Complexes. *chem. Lett.* **2015**, *44*, 563–565, doi:10.1246/cl.141203.
22. Constable, E.C.; Neuburger, M.; Rösel, P.; Schneider, G.E.; Zampese, J.A.; Housecroft, C.E.; Monti, F.; Armaroli, N.; Costa, R.D.; Ortí, E. Ligand-Based Charge-Transfer Luminescence in Ionic Cyclometalated Iridium(III) Complexes Bearing a Pyrene-Functionalized Bipyridine Ligand: A Joint Theoretical and Experimental Study. *Inorg. Chem.* **2013**, *52*, 885–897, doi:10.1021/ic302026f.
23. Denisov, S.A.; Cudré, Y.; Verwilst, P.; Jonusauskas, G.; Marín-Suárez, M.; Fernández-Sánchez, J.F.; Baranoff, E.; McClenaghan, N.D. Direct Observation of Reversible Electronic Energy Transfer Involving an Iridium Center. *Inorg. Chem.* **2014**, *53*, 2677–2682, doi:10.1021/ic4030712.
24. Medina-Rodríguez, S.; Denisov, S.A.; Cudré, Y.; Male, L.; Marín-Suárez, M.; Fernández-Gutiérrez, A.; Fernández-Sánchez, J.F.; Tron, A.; Jonusauskas, G.; McClenaghan, N.D.; et al. High Performance Optical Oxygen Sensors Based on Iridium Complexes Exhibiting Interchromophore Energy Shuttling. *Analyst* **2016**, *141*, 3090–3097, doi:10.1039/C6AN00497K.
25. Jin, Z.; Qi, S.; Guo, X.; Tian, N.; Hou, Y.; Li, C.; Wang, X.; Zhou, Q. Smart Use of "Ping-Pong" Energy Transfer to Improve the Two-Photon Photodynamic Activity of an Ir(III) Complex. *Chem. Commun.* **2020**, *56*, 2845–2848, doi:10.1039/C9CC09763E.
26. Lu, Y.; Wang, J.; McGoldrick, N.; Cui, X.; Zhao, J.; Caverly, C.; Twamley, B.; Ó Máille, G.M.; Irwin, B.; Conway-Kenny, R.; et al. Iridium(III) Complexes Bearing Pyrene-Functionalized 1,10-Phenanthroline Ligands as Highly Efficient Sensitizers for Triplet–Triplet Annihilation Upconversion. *Angewandte Chemie International Edition* **2016**, *55*, 14688–14692, doi:10.1002/anie.201608442.
27. Lu, Y.; McGoldrick, N.; Murphy, F.; Twamley, B.; Cui, X.; Delaney, C.; Máille, G.M.Ó.; Wang, J.; Zhao, J.; Draper, S.M. Highly Efficient Triplet Photosensitizers: A Systematic Approach to the Application of IrIII Complexes Containing Extended Phenanthrolines. *Chemistry – A European Journal* **2016**, *22*, 11349–11356, doi:10.1002/chem.201601534.
28. Lu, Y.; Conway-Kenny, R.; Wang, J.; Cui, X.; Zhao, J.; Draper, S.M. Exploiting Coumarin-6 as Ancillary Ligands in 1,10-Phenanthroline Ir(III) Complexes: Generating Triplet Photosensitisers with High Upconversion Capabilities. *Dalton Trans.* **2018**, *47*, 8585–8589, doi:10.1039/C8DT00231B.
29. Seth, S.K.; Purkayastha, P. Unusually Large Singlet Oxygen (1O2) Production by Very Weakly Emissive Pyrene-Functionalized Iridium(III) Complex: Interplay between Excited 3ILCT/3IL and

3MLCT States. *European Journal of Inorganic Chemistry* **2020**, *2020*, 2990–2997, doi:10.1002/ejic.202000442.
30. Zhu, X.; Cui, P.; Kilina, S.; Sun, W. Multifunctional Cationic Iridium(III) Complexes Bearing 2-Aryloxazolo[4,5-f][1,10]Phenanthroline (N^N) Ligand: Synthesis, Crystal Structure, Photophysics, Mechanochromic/Vapochromic Effects, and Reverse Saturable Absorption. *Inorg. Chem.* **2017**, *56*, 13715–13731, doi:10.1021/acs.inorgchem.7b01472.
31. Olumba, M.E.; O'Donnell, R.M.; Rohrabaugh, T.N.J.; Teets, T.S. Triplet–Triplet Energy Transfer in Bis-Cyclometalated Iridium Complexes with Pyrene-Substituted Isocyanides. *Inorg. Chem.* **2023**, *62*, 13702–13711, doi:10.1021/acs.inorgchem.3c00457.
32. Fan, S.; Zong, X.; Shaw, P.E.; Wang, X.; Geng, Y.; Smith, A.R.G.; Burn, P.L.; Wang, L.; Lo, S.-C. Energetic Requirements of Iridium(III) Complex Based Photosensitisers in Photocatalytic Hydrogen Generation. *Phys. Chem. Chem. Phys.* **2014**, *16*, 21577–21585, doi:10.1039/C4CP02997F.
33. Liu, B.; Lystrom, L.; Cameron, C.G.; Kilina, S.; McFarland, S.A.; Sun, W. Monocationic Iridium(III) Complexes with Far-Red Charge-Transfer Absorption and Near-IR Emission: Synthesis, Photophysics, and Reverse Saturable Absorption. *European Journal of Inorganic Chemistry* **2019**, *2019*, 2208–2215, doi:10.1002/ejic.201900156.
34. Liu, B.; Lystrom, L.; Kilina, S.; Sun, W. Tuning the Ground State and Excited State Properties of Monocationic Iridium(III) Complexes by Varying the Site of Benzannulation on Diimine Ligand. *Inorg. Chem.* **2017**, *56*, 5361–5370, doi:10.1021/acs.inorgchem.7b00467.
35. Yi, X.; Yang, P.; Huang, D.; Zhao, J. Visible Light-Harvesting Cyclometalated Ir(III) Complexes with Pyreno[4,5-d]Imidazole *C^N* Ligands as Triplet Photosensitizers for Triplet–Triplet Annihilation Upconversion. *Dyes and Pigments* **2013**, *96*, 104–115, doi:10.1016/j.dyepig.2012.07.020.
36. Shi, C.; Sun, H.; Jiang, Q.; Zhao, Q.; Wang, J.; Huang, W.; Yan, H. Carborane Tuning of Photophysical Properties of Phosphorescent Iridium(III) Complexes. *Chem. Commun.* **2013**, *49*, 4746–4748, doi:10.1039/C3CC40996A.
37. Dragonetti, C.; Falciola, L.; Mussini, P.; Righetto, S.; Roberto, D.; Ugo, R.; Valore, A.; De Angelis, F.; Fantacci, S.; Sgamellotti, A.; et al. The Role of Substituents on Functionalized 1,10-Phenanthroline in Controlling the Emission Properties of Cationic Iridium(III) Complexes of Interest for Electroluminescent Devices. *Inorg. Chem.* **2007**, *46*, 8533–8547, doi:10.1021/ic700414z.
38. Takizawa, S.; Pérez-Bolívar, C.; Anzenbacher Jr., P.; Murata, S. Cationic Iridium Complexes Coordinated with Coumarin Dyes – Sensitizers for Visible-Light-Driven Hydrogen Generation. *European Journal of Inorganic Chemistry* **2012**, *2012*, 3975–3979, doi:10.1002/ejic.201200474.
39. Zhao, Q.; Liu, S.; Shi, M.; Li, F.; Jing, H.; Yi, T.; Huang, C. Tuning Photophysical and Electrochemical Properties of Cationic Iridium(III) Complex Salts with Imidazolyl Substituents by Proton and Anions. *Organometallics* **2007**, *26*, 5922–5930, doi:10.1021/om700623j.
40. Olumba, M.E.; O'Donnell, R.M.; Rohrabaugh, T.N.Jr.; Teets, T.S. Panchromatic Excited-State Absorption in Bis-Cyclometalated Iridium Isocyanide Complexes. *Inorg. Chem.* **2022**, *61*, 19344–19353, doi:10.1021/acs.inorgchem.2c03136.
41. Genoni, A.; Chirdon, D.N.; Boniolo, M.; Sartorel, A.; Bernhard, S.; Bonchio, M. Tuning Iridium Photocatalysts and Light Irradiation for Enhanced CO2 Reduction. *ACS Catal.* **2017**, *7*, 154–160, doi:10.1021/acscatal.6b03227.
42. Yadav, A.K.; Singh, V.; Kushwaha, R.; Kunwar, A.; Koch, B.; Banerjee, S. Anticancer Potential of Polypyridyl-Based Ir(III)-Coumarin 6 Conjugates under Visible Light and Dark. *Inorganic Chemistry Communications* **2025**, *175*, 114184, doi:10.1016/j.inoche.2025.114184.
43. Romero-Castellón, I.; Markova, L.; Piernas-Muñoz, M.J.; Kostrhunova, H.; Kasparkova, J.; Janiak, C.; Alberto, M.E.; Francés-Monerris, A.; Ruiz, J.; Brabec, V. Photocatalytic Arylterpyridine Iridium(III) Complexes Trigger Oncosis in 2D and 3D Cancer Cell Models via NADH Oxidation. *Inorg. Chem. Front.* **2026**, *13*, 2045–2063, doi:10.1039/D5QI02205C.
44. Kuang, S.; Sun, L.; Zhang, X.; Liao, X.; Rees, T.W.; Zeng, L.; Chen, Y.; Zhang, X.; Ji, L.; Chao, H. A Mitochondrion-Localized Two-Photon Photosensitizer Generating Carbon Radicals Against

Hypoxic Tumors. *Angewandte Chemie International Edition* **2020**, *59*, 20697–20703, doi:10.1002/anie.202009888.

45. Singh, V.D.; Paitandi, R.P.; Dwivedi, B.K.; Singh, R.S.; Pandey, D.S. Cyclometalated Ir(III) Complexes Involving Functionalized Terpyridine-Based Ligands Exhibiting Aggregation-Induced Emission and Their Potential Applications in CO2 Detection. *Organometallics* **2018**, *37*, 3827–3838, doi:10.1021/acs.organomet.8b00520.
46. Liu, L.; Chen, J.; Wang, M.-M.; Huang, Y.; Qian, Y.; Xue, X.; Su, Z.; Liu, H.-K. The Cyclometalated Iridium (III) Complex Based on 9-Anthracenecarboxylic Acid as a Lysosomal-Targeted Anticancer Agent. *Journal of Inorganic Biochemistry* **2022**, *235*, 111913, doi:10.1016/j.jinorgbio.2022.111913.
47. Liu, X.; Dai, P.; Gu, T.; Wu, Q.; Wei, H.; Liu, S.; Zhang, K.Y.; Zhao, Q. Cyclometalated Iridium(III) Complexes Containing an Anthracene Unit for Sensing and Imaging Singlet Oxygen in Cellular Mitochondria. *Journal of Inorganic Biochemistry* **2020**, *209*, 111106, doi:10.1016/j.jinorgbio.2020.111106.
48. Redrado, M.; Benedi, A.; Marzo, I.; Gimeno, M.C.; Fernández-Moreira, V. Dual Emissive Ir(III) Complexes for Photodynamic Therapy and Bioimaging. *Pharmaceutics* **2021**, *13*, doi:10.3390/pharmaceutics13091382.
49. Ghosh, S.; Paira, P. Instigating Visible Light Inspired DNA Impairment by ROS Harvesting Ir(III)-Cyclometallated Imidazophenanthroline Complexes Against MDA-MB-231 Cells. *European Journal of Inorganic Chemistry* **2025**, *28*, e202400769, doi:10.1002/ejic.202400769.
50. Gao, T.-B.; Qu, Z.-Z.; Tang, Z.; Cao, D.-K. Cyclometalated Ir(III) Complexes Incorporating a Photoactive Anthracene-Based Ligand: Syntheses, Crystal Structures and Luminescence Switching by Light Irradiation. *Dalton Trans.* **2017**, *46*, 15443–15450, doi:10.1039/C7DT02967E.
51. Yuan, Q.-Z.; Fan, Q.; Lv, H.; Chen, W.-W.; Yang, X.-X.; Cao, D.-K.; Wen, J. Two Anthracene-Based Ir(III) Complexes [Ir(Pbt)2(Aip)]Cl and [Ir(Pbt)2(Aipm)]Cl: Relationship between Substituent Group and Photo-Oxidation Activity as Well as Photo-Oxidation-Induced Luminescence. *Inorg. Chem.* **2020**, *59*, 17071–17076, doi:10.1021/acs.inorgchem.0c02279.
52. Kuang, S.; Wei, F.; Karges, J.; Ke, L.; Xiong, K.; Liao, X.; Gasser, G.; Ji, L.; Chao, H. Photodecaging of a Mitochondria-Localized Iridium(III) Endoperoxide Complex for Two-Photon Photoactivated Therapy under Hypoxia. *J. Am. Chem. Soc.* **2022**, *144*, 4091–4101, doi:10.1021/jacs.1c13137.
53. Choroba, K.; Palion-Gazda, J.; Penkala, M.; Rawicka, P.; Machura, B. Tunability of Triplet Excited States and Photophysical Behaviour of Bis-Cyclometalated Iridium(III) Complexes with Imidazo[4,5-f][1,10]Phenanthroline. *Dalton Trans.* **2024**, *53*, 17934–17947, doi:10.1039/D4DT01996B.
54. Jin, C.; Liu, J.; Chen, Y.; Li, G.; Guan, R.; Zhang, P.; Ji, L.; Chao, H. Cyclometalated Iridium(III) Complexes with Imidazo[4,5-f][1,10]Phenanthroline Derivatives for Mitochondrial Imaging in Living Cells. *Dalton Trans.* **2015**, *44*, 7538–7547, doi:10.1039/C5DT00467E.
